# On the Noise Complexity in an Optical Motion Capture Facility

**DOI:** 10.3390/s19204435

**Published:** 2019-10-13

**Authors:** Przemysław Skurowski, Magdalena Pawlyta

**Affiliations:** 1 Institute of Informatics, Silesian University of Technology, Akademicka 16, 44-100 Gliwice, Poland; 2 Polish-Japanese Academy of Information Technology, Koszykowa 86, 02-008 Warsaw, Poland; Magdalena.Pawlyta@polsl.pl

**Keywords:** motion capture, evaluation, noise modelling, noise color, Allan variance, simulated annealing, ant colony optimization

## Abstract

Optical motion capture systems are state-of-the-art in motion acquisition; however, like any measurement system they are not error-free: noise is their intrinsic feature. The works so far mostly employ a simple noise model, expressing the uncertainty as a simple variance. In the work, we demonstrate that it might be not sufficient and we prove the existence of several types of noise and demonstrate how to quantify them using Allan variance. Such a knowledge is especially important for using optical motion capture to calibrate other techniques, and for applications requiring very fine quality of recording. For the automated readout of the noise coefficients, we solve the multidimensional regression problem using sophisticated metaheuristics in the exploration-exploitation scheme. We identified in the laboratory the notable contribution to the overall noise from white noise and random walk, and a minor contribution from blue noise and flicker, whereas the violet noise is absent. Besides classic types of noise we identified the presence of the correlated noises and periodic distortion. We analyzed also how the noise types scale with an increasing number of cameras. We had also the opportunity to observe the influence of camera failure on the overall performance.

## 1. Introduction

Synthesis and analysis of human motion is an active research area with a plurality of applications in biomechanics and entertainment [1]. Contemporary technologies allow capturing and processing movement (Mocap) with high realism and accuracy; however, they are not error-proof. Various methods were proposed for motion acquisition, yet the optical motion capture (OMC) technique, based on tracking of retro-reflective markers in IR images is considered the gold standard in this field of research. It outperforms other techniques and it has been used for verification of the other technologies: inertial [2,3] or optical [4]. OMC is also considered to be a reference motion acquisition for the applications of other Mocap technologies in such demanding areas as medical [5,6,7] or space research [8].

The uncertainty in optical motion capture systems depends on numerous factors, such as type and amount of used cameras, their physical setup, and mounting, marker size, environmental conditions such as air temperature or humidity, camera noise, and quality of the calibration of the motion camera in the motion capture system. Although almost all these factors can be controlled by re-calibration of the system or ensuring constant environmental conditions, the noise present in the cameras is an inevitable factor that cannot be easily neglected or removed.

In this article we characterize the types and levels of noise in three types of Vicon Motion Capture Camera: MX-T40, Bonita10 and Vantage 5. We use Allan variance (AVAR) [9] which is a handy tool for identification and evaluation of noise types. We propose how to address the non-trivial regression problem of ADEV curve, by matching it with the component functions using metaheuristics: simulated annealing (SA) and ant colony optimization (ACO). Moreover, thanks to the observed malfunction of one of the devices we were able to demonstrate that the proposed approach can be used for quite complex cases of correlated and periodic distortions.

The proposed usage of Allan variance has several advantages over the classical statistical approach or spectral analysis. It works when the simple statistics fails, it is adequate for the noise quantification when the measurements are correlated. It has clear graphical interpretation, which allows for identification and quantifying several different categories of noise at once. Last but not least it does not require a template: it is based on the assumption that the signal is constant and any variation is noise actually.

The need for obtaining cautious characteristics of the OMC systems is severalfold. The key rationale is that OMC systems are employed as a reference for the other Mocap techniques. Requirements of the reference systems for calibration of measurement devices are high, demanding the reference precision to outperform a system under tests significantly. Moreover, the presence of the non-standard types of noise can falsify conventional measures of uncertainty in such a system. Finally, the knowledge of occurrence and amplitude of certain classes of noise can be useful for diagnosing the facility and reducing the noise sources (e.g., long term environmental influence).

The article is organized as follows: in Section 2 we provide theoretical background and we demonstrate simple variance-based noise quantification with the precaution that it might be not satisfactory; we introduce Allan variance as an alternative. In Section 3 we describe the experiment—laboratory setup and procedures—followed by an algorithm for parameters estimation description and comments on unexpected phenomena observed during the experiment. Section 4 discloses the experimental results and their analysis followed by a discussion of results. Section 5 summarizes the article and provides ideas for future work.

## 2. Background

### 2.1. Previous Works

The accuracy and precision in different OMCs were subject to analysis in several works [10,11,12,13]. In those works, the most frequently studied OMCs are one of Vicon System (MX, Bonita, and V-series), or the OptiTrack system. Regardless of the system used, the authors of these studies agree that the most important factor that influences the data is camera calibration. Camera calibration originates from photogrammetry [14], it relies on positioning the cameras in a virtual 3D space so that they correspond to the cameras positions in the laboratory. This position and several (minimum two) 2D camera projections of markers are used to reconstruct markers in 3D space [15]. The calibration quality is determined using average re-projection error. This is the mean distance between the 2D image of the markers on camera and 3D reconstructions of those markers projected back to the camera’s sensor in pixels.

Early works modeled the noise in frequency-based fashion for signal processing needs. Values of a high frequency were considered to be noise that needed to be removed. They were identified or as residuals from the ‘right’ motion modeled by slowly varying curves (low-order polynomials, splines, or Fourier series of low order) [16,17], or by conventional Butterworth filtering [18,19], where the cut-off frequency was identified by the lack of autocorrelation in the filtered-out residuals.

Windolf et al. [11] reported that performance of OMC strongly depends on their individual setup and that accuracy and precision should be determined for an individual laboratory installation. They tested both accuracy as a root-mean-square (RMS) error from ground truth and precision as a standard deviation of measured positions in a four camera Vicon 460 system. As a ground truth they employed a custom-built robot mounted L-shaped template. They verified the influence of changing the camera setup, calibration volume, marker size and lens filter application. In the best case they report 63 ± 5 μm accuracy and 15 μm precision.

In another study, Jensenius et al. [13] tested two OMC systems: Optitrack and Qualisys. They used constancy of position as a quality criterion and identified marker position drifting over the time. They measured drifting velocity (in mm/s) and drifting range (in mm) that identifies volume of uncertainty for marker position. They also emphasize role of proper calibration for the performance of OMC, and coverage of the area within the calibration procedure.

In the work of Carse et al. [12], three optical 3D motion analysis systems were compared, one of which was a new low-cost system (Optitrack), and two which were considerably more expensive (Vicon 612 and Vicon MX). They used a rigid cluster of markers and measured inter-marker distance and its standard deviation (SD) as a quality criterion for a walking task in an unknown, but adequately large volume. They reached SD values between 0.11–3.7 mm depending on the OMC system.

Results confirming high quality position measurement, using Vicon MX with 5 Vicon F-40 cameras, were obtained in the work by Yang et al. [20]. They considered whether the OMC could be used for the subtle bone deformation during exercises; the task required accuracy better than 20 μm. As a test template they used markers mounted on the computer numerical controlled (CNC) milling machine with 1 μm spatial resolution. They tested influence of marker size for cameras located very close to the observed, quite small, volume (0.4 × 0.3 × 0.3 m). They confirmed that it is possible to achieve the RMSE accuracy and precision to be 1.2–1.8 μm and 1.5–2.5 μm respectively.

Eichelberger et al. [10] investigated the influence of various recording parameters on the accuracy using Vicon Bonita cameras. These are the number of cameras (6, 8 and 10), measurement height (foot, knee and hip) and movement (static and dynamic). All these affected the system accuracy significantly.

Another notable work was conducted by Merriaux et al. [21]. They performed two experimental error estimations in 8 Vicon T40 camera OMC in moderate volume 2 × 1.5 × 1 m. They used two sophisticated robotic templates for static and dynamic (fast rotating blade) cases. In the static case, the estimated errors are mean absolute error (MAE) 0.15 mm for accuracy and RMSE of 0.015 mm for precision. In the dynamic case, the observed accuracy was larger, yet still satisfying, it achieved values between 0.3 mm to <2 mm. They demonstrated also that it depends on the object velocity and sampling frequency.

Slightly different, yet interesting study on noise [22] involved aquatic OMC based on Vicon T40 cameras, where the scene was a water-filled tank, cameras are located externally in dry locations and the markers made of dedicated reflective tape (SOLAS) are submerged. They demonstrated no significant difference in accuracy and precision due to various mediums in the optical path.

The technological side of an OMC is not the only source of distortions in the system. In the work of Capozzo et al. [23], the mechanics of markers placed on the skin was emphasized as a source of distortions in OMC. Further, it has been practically considered in the work of Alexander and Andriacchi [24], where skin motion-based distortions were suppressed. Clusters of marker positions were observed in a non-disclosed OMC. Bone orientation was estimated and evaluated, but marker positions were also analyzed; however, they were not the main quality criterion. They were able to reduce marker location error from 0.025 to 0.008 cm and the average bone orientation error from 0.370 to 0.083 degrees.

Various requirements towards the system uncertainty were specified in the literature. They depend on the applications and recorded tasks [1]. Some applications may require very high accuracy and precision achievable in a small volume, whereas most of the motion capture labs need larger observed volume at the expense of quality for practical purposes [25]. Moreover, due to various process dynamics and to avoid motion blur, some tasks may require much higher sampling frequency than 100–120 Hz, which is typically used. Detailed handwriting analysis for subtle symptoms of cognitive issues requires small volume but high accuracy and frequent sampling [26], whereas for some sports activities the volumes might be huge but the accuracy of meters is enough [27]. Even the same area of applications may require very different parameters of motion acquisition. Exemplarily, in medical applications, the aforementioned [20] bone deformation acquisition needs high accuracy in small volume; whereas the behavioral study of the surgery staff [28], which took place in 6 × 6 m operating room, resulted in 10% of recordings, which were inconclusive for identification of a subject.

### 2.2. Simple Preliminary Gaussian Model

**Figure 1:**
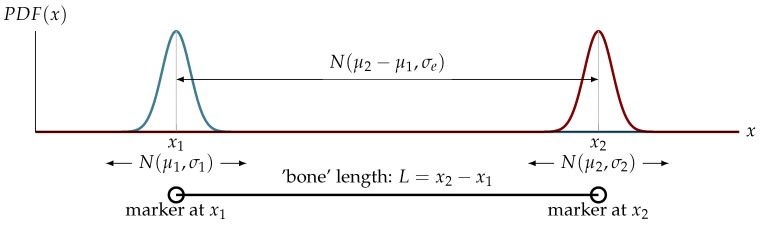
Schematic of situation and corresponding theoretic probability—two markers at *x*_1_ and *x*_2_ identifying a single rigid body (bone) of length *l*.

Locating markers in a scene is a continuous process occurring frame-by-frame at the requested sampling frequency. The measurement of the location of a marker can be presumed to be an actual location signal plus additive Gaussian white noise, consequently, locating of each marker location is an independent statistical process. One dimensional case, as depicted in Figure 1, can be described with normal probability density function:

(1) $$loc\left(M_{k}\right)\approx x_{k}=N\left(\mu_{k},\sigma_{k}\right),$$

where: $loc(M_{k})$ denotes actual location of *k*th marker in a scene. N(·) denotes normal (Gaussian) distribution, which for real location at $x_{k}$ is estimated as a mean $\mu_{k}$, and standard deviation $\sigma_{k}$, that (at best) should be common for all the same markers (of a same size).

The typical uncertainty analysis in measurements employs two factors accuracy and precision [29]—the accuracy that describes how close the estimate $\mu_{k}$ is to actual location $x_{k}$ and describes the systematic error, whereas $\sigma_{k}$ reflects the precision of measurement and describes random part of the error.

Extending the estimation of a marker model to the estimation of a length (*L*) of a bone, it yields a difference of double marker location measurements, hence its probability density function is described:

(2) $$length\left(x_{1},x_{2}\right)\approx PDF\left(L\right)=\left|N\left(\mu_{2},\sigma \right)-N\left(\mu_{1},\sigma \right)\right|=N\left(\mu_{e},\sigma_{e}\right),$$

where: $\mu_{e}=\left|x_{2}-x_{1}\right|$—expected (in common sense) mean value, $\sigma_{e}$—expected standard deviation, which might take different forms, depending on the case:

- $\sigma_{A}=\sigma \sqrt{2}$—for two identical ($\sigma_{1}=\sigma_{2}$), independent variances (covariance $\sigma_{12}=0$),
- $\sigma_{B}=\sqrt{\sigma_{1}^{2}+\sigma_{1}^{2}}$—for two different ($\sigma_{1}\neq \sigma_{2}$), independent variances ($\sigma_{12}=0$),
- $\sigma_{C}=\sqrt{\sigma_{1}^{2}+\sigma_{2}^{2}-2\sigma_{12}}$—for two different ($\sigma_{1}\neq \sigma_{2}$), correlated variances ($\sigma_{12}\neq 0$).

**Figure 2:**
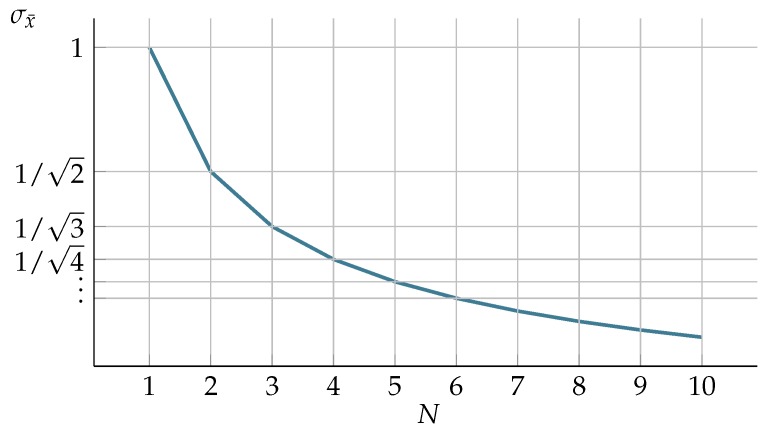
Standard error for estimating actual position with increasing number of samples.

The number of cameras used for position reconstruction is another factor that has a significant influence on the uncertainty of measured position. In the system which takes multiple samples of the measured value (such as position) and results in a mean value of these, the perceived precision can be described as standard error (SE) [30]. In our case, the increasing number of cameras used for reconstruction could be considered to be taking successive samples of the position. With the increasing number of samples, the SE value falls as the variability of measured value reduces. SE calculation is based on standard deviation:

(3) $$\sigma_{\bar{x}}=\frac{\sigma }{\sqrt{N}},$$

where: *N* is several observations, *σ*—standard deviation. This theoretic, quasi-hyperbolic relationship is depicted in Figure 2. Such a description is just a kind of approximation of the uncertainty variation in the multicamera triangulation process, since we do not exactly know all the nuances of implementation by OMC vendor; furthermore it does not take into consideration spatial location of cameras.

The other issue of the error quantification is the lack of reliable ground truth. The Vicon systems return their results with 1/100 mm resolution, though it is known (see Section 2) that the actual accuracy of OMCs in real installations is lower. Yet still, the uncertainty of reference has to be much better than the tested system. According to the meteorology standards, reference uncertainty should be smaller between 4 and 10 times than the system under test [30]. It is difficult to obtain necessary physical template (like the T-frame) manufactured with precision and accuracy sufficient to reliably calibrate OMCs. For this reason, it is hardly feasible to evaluate the accuracy (bias) of the length estimation with mean values without sophisticated equipment. Fortunately, this aspect is of lesser concern as it describes the systematic error, which is easy to compensate.

However, all above considerations would not be enough if the input location measurements are correlated. According to metrology guidelines [29] simple experimental mean or standard deviation are not adequate to describe the uncertainty in the system with correlated noises. In such a situation a dedicated tool, namely Allan variance, is recommended.

### 2.3. Allan Variance

Allan variance (AVAR) a two-sample variance and its square root—Allan deviation (ADEV) are statistical descriptors that were developed for the evaluation of the stability of the time and oscillation in clocks. A notable advantage of this approach that there is no need to provide reference value or ground truth.

Presently, the measure is effectively used for quantifying the noises in the measurement of other quantities [31,32], but it is particularly useful for evaluation of inertial motion capture sensors [33,34]. Allan variance [9] is defined as:

(4) $$\begin{array}{c} \sigma_{y}^{2}\left(\tau \right)=\frac{1}{2}\langle {\left(y\left(t+\tau \right)-y\left(t\right)\right)}^{2}\rangle , \end{array}$$

where τ is the time intersample spacing, 〈·〉 denotes expected value.

**Figure 3:**
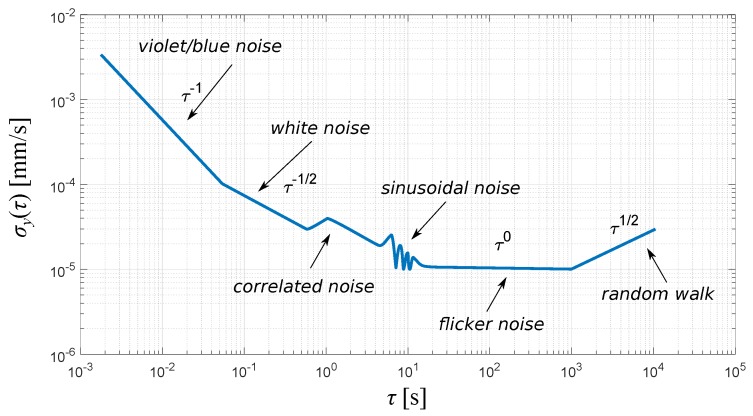
Schematic view on Allan Deviation log-log plot (axis values are for illustrative proposes).

The AVAR analysis consists of identifying the linear parts of certain slopes of the log-log plot of *τ* steps versus ADEV (square root of AVAR). It is demonstrated in the schematic ADEV plot in Figure 3. It is a highly beneficial advantage of the AVAR noise quantification over the power spectral density (PSD), which has the capability not to clutter different noise processes and to precisely discriminate several types at once. However, there are also disadvantages. AVAR is sensitive to the outliers and requires considering outlier cleaning to obtain reliable results. The second issue is a necessity to record quite a long sequence for the analysis of a longer term processes.

The conventional types of noise can be identified by their PSD distribution with the power law and respective ADEV slopes [35]. The ’color’ is given as power relation with respect to frequency ($S\left(f\right)\propto 1/f^{\alpha }$). Therefore, overall noise characteristics, comprising different basic noise types are:

(5) $$S\left(f\right)=\sum_{\alpha }h_{\alpha }f^{\alpha }.$$

It corresponds to:

(6) $$\sigma_{y}^{2}\left(\tau \right)\approx \sum_{\tau }h_{\alpha }K_{\alpha }\tau^{\mu },$$

which for a conventional set of noises yields:

(7) $$\sigma_{y}^{2}\left(\tau \right)\approx Ah_{-2}\tau +Bh_{-1}+Ch_{0}\tau^{-1}+\left(Dh_{1}+Eh_{2}\right)\tau^{-2}.$$

Conventional (color) noise types are gathered in Table 1,

where $f_{h}$ is bandwidth limit for the measurement system. A..E respective scaling factors $K_{\alpha }$.

Additionally, two complex distortions, exponentially correlated (Markovian) and sinusoidal, can be identified using Allan variance [36]. The Markovian noise is visible in the Allan deviation plot as a single ’bump’ with slopes $\pm \frac{1}{2}$. Periodic (sinusoidal) distortion is represented in respective plot as a decaying series of bumps with left-sided slope 1 and right side bump series with constant envelope of a slope −1; however, it is the only case that is more convenient to be observed and to analyze the distortion in the Fourier spectral domain.

Correlated noise PSD is given as:

(8) $$S_{c}\left(f\right)=\frac{{\left(q_{c}T_{c}\right)}^{2}}{1+{\left(2\pi fT_{c}\right)}^{2}},$$

and corresponding Allan variance has a form:

(9) $$\sigma_{c}^{2}\left(\tau \right)=\frac{{\left(q_{c}T_{c}\right)}^{2}}{\tau }\left(1-\frac{T_{c}}{2\tau }\left(3-4e^{-\frac{\tau }{T_{c}}}+e^{-\frac{2\tau }{T_{c}}}\right)\right)$$

where: $q_{c}$ is the noise amplitude, $T_{c}$ is the correlation time.

Sinusoidal noise PSD has a form of two peaks, modeled with Dirac delta:

(10) $$S_{s}\left(f\right)=\frac{1}{2}A_{s}^{2}\left(\delta \left(f-f_{0}\right)+\delta \left(f+f_{0}\right)\right),$$

and respective Allan variance form:

(11) $$\sigma_{s}^{2}\left(\tau \right)=A_{s}^{2}\left(\frac{\sin^{2}\left(\pi f_{0}\tau \right)}{\pi f_{0}\tau }\right),$$

here: $A_{s}$ is the amplitude, $f_{0}$ is the frequency, δ(·) is Dirac delta peak.

## 3. Materials and Methods

### 3.1. Environment

The experimental setup was employed in the Human Motion Laboratory (HML) at the Research and Development Centre of Polish-Japanese Academy of Information Technology in Bytom (http://bytom.pja.edu.pl/laboratorium/laboratorium-hml-analizy-ruchu-czlowieka/). The motion system used in this laboratory consists of a total of 30 Vicon Motion cameras of three different types:

- 10 Vicon MX-T40,
- 10 Vicon Bonita10,
- 10 Vicon Vantage V5.

**Figure 4:**
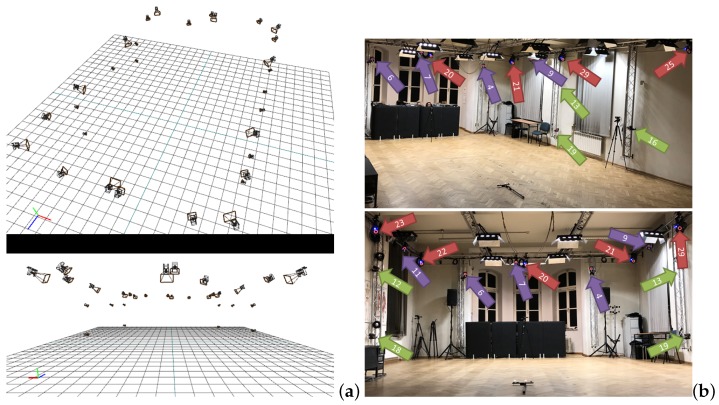
Camera locations: (**a**) Schematic view in Vicon Blade software, (**b**) actual setup in HML. Color denotes camera series: violet—Vantage, green—Bonita, red—T40.

These cameras can record data independently or can be integrated into one larger system with capture volume 9 m × 5 m × 3 m. To minimize the impact of external interference like infrared interference from sunlight or vibrations, all windows are permanently darkened and cameras are mounted on scaffolding instead of tripods (as is shown in Figure 4) The basic information and main differences between used cameras are shown in Table 2.

### 3.2. Data Capture

**Figure 5:**
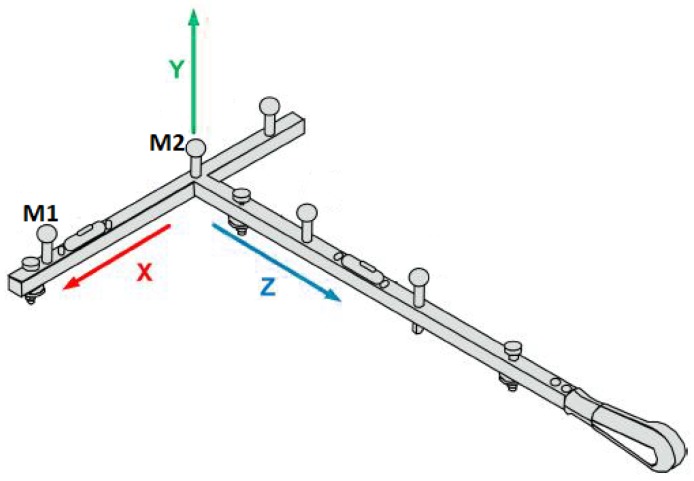
Vicon calibration wand schema (T-frame).

For the noise analysis needs, a special, nine-hour recording of the two 14 mm markers from the calibration T-frame template (wand) was made. The duration was chosen to reveal the influence of longer term processes, such as a random walk, and to be on par with the length of a normal daily working hours. The wand (demonstrated in Figure 5) was placed in the center of motion capture volume. The three other markers were removed. Data was recorded simultaneously by all the 30 cameras at 120 Hz in standard Vicon software (Vicon Blade version 3.3.1). The XYZ coordinate system was by default oriented according to the T-frame as it is depicted in Figure 5. Camera calibration was made once with all thirty cameras according to the standard Vicon procedure. The reprojection error for this session, for all the cameras was less than 0.2 pix—mean error for Bonita −0.1946 pix; Vantage −0.1891 pix; T40 −0.1535 pix as reported by the software after the calibration procedure. Additionally, in order to minimize the environmental noise, laboratory technicians were not present in the room during this recording. After the system calibration, all the necessary operations and supervision (start and stop record, system status verification, etc.) were done remotely.

### 3.3. Data Processing

In the post-processing stage in Vicon Blade (Version 3.3.1) software, markers were reconstructed and labeled only, no other filtering or processing was used. This stage was done several times, separately for each camera configuration (including different numbers of cameras of each type). Reconstruction settings were set to the default, for each camera type except the initial set of 2 cameras of each type, where it was required to override the demand of marker visibility by three cameras at least. In this trial, the parameter *‘Minimum Cameras to Start Trajectory’* had to be set to 2. All those data were used to create the few datasets, containing several realizations of the same sequence:

- Data set 1: based on all cameras
- Data set 2: based on T40 cameras
- Data set 3: based on Bonita cameras
- Data set 4: based on Vantage cameras

The data sets 2–4 consists of 7 trials, in which a different number of cameras used for 3D markers reconstruction (Table 3). The location of each camera is shown in Figure 4. To characterize the noise in different camera type, in all datasets the x,y,z trajectories of both markers and their Euclidean (Equation (12)) distance were analyzed.

(12) $$\begin{array}{c} L=d\left(M_{1},M_{2}\right)=\sqrt{{\left(x_{1}+x_{2}\right)}^{2}+{\left(y_{1}+y_{2}\right)}^{2}+{\left(z_{1}+z_{2}\right)}^{2}} \end{array}$$

Processing operations in Vicon Blade were limited to 3D reconstruction of marker trajectories and exporting the data to the .c3d file format. Further filtering, analysis, and processing of data were done with Matlab (Version R2016b).

### 3.4. Noise Parameters Estimation

For the computing of AVAR from the experimental data we used an implementation by Czerwinski [32]. It implements various AVAR versions, including overlapping estimator, that we chose to use as it is more stable and boundary error prone than conventional one.

The notable advantage of Allan deviation plots is their simple visual interpretation. Moreover, identification of complex—sinusoidal or correlated—distortions is possible just by visual inspection [33] for the presence of bumps in the plot. Another beneficial feature is the ability to estimate the parameters by simple line or poly-line matching to log-log plot [34]. However, straightforward distinguishing between blue and violet noises is not possible in such a case—to obtain these phase dependant noises it would be necessary to employ a much slower variant—modified AVAR estimation.

The method for the noise parameters readout from the ADEV curve, used in this research was proposed by Vernotte et al. in [37]. The model employs minimization of weighted least squares (WLS). As it was demonstrated in [31], such an LS model even allows to identify blue and violet noises which are represented jointly by $\tau^{-2}$ component.

The weighted LS is represented as following minimization problem that reduces the weighted error between measured AVAR values ${\hat{\sigma }}_{y}^{2}\left(\tau \right)$ and a sum of estimated components $\sigma_{i}^{2}\left(\tau \right)$-s:

(13) $$\hat{H}=\underset{h_{-2},\ldots ,h_{2},q_{c},T_{c},A_{s},f_{o}\geq 0}{arg min}\left(\sum_{\tau }{\left[\frac{1}{{\hat{\sigma }}_{y}^{2}\left(\tau \right)}\left({\hat{\sigma }}_{y}^{2}\left(\tau \right)\;-\sum_{i=\{\begin{array}{c} v,b,w,\\ f,r,c,s \end{array}\}}\sigma_{i}^{2}\left(\tau \right)\right)\right]}^{2}\right).$$

**Figure 6:**
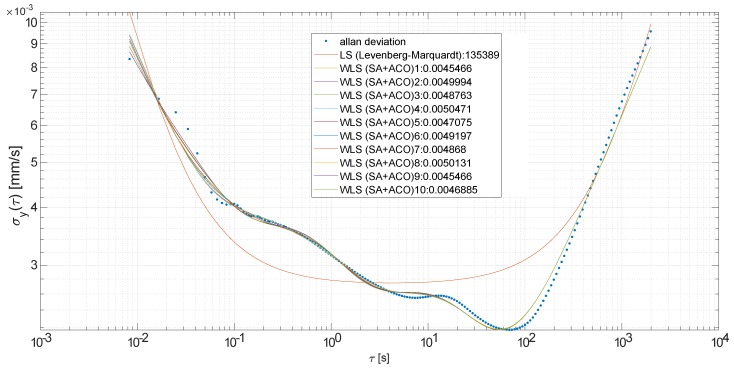
Regression results for an excerpt from the experimental data, demonstrates complex ADEV curve with periodic and correlated components and 10 starts of WLS compared with ordinary LS.

Obtaining reasonable results for such a complexand multideimensional non-linear model is a challenging issue. Therefore, we followed roughly a multi-start hybrid algorithm proposed in [38]—multi-start simulated annealing followed by local minimum search, where multiple starts prevent dependence on the initialization. It follows exploration-exploitation scheme, in the first stage simulated annealing (SA), known for avoiding of getting stuck in local minimum, finds solution close to global optimum, which is then refined by local pattern search—for the latter we propose to use ant colony optimization (ACO), specifically the ${\mathrm{ACO}}_{R}$ variant for continuous domains [39]. Our additional modification is in the initialization stage of the ACO solver, which is at start populated with values jittered around the solution returned by the SA stage. Exemplary regression results are visually demonstrated in Figure 6, compared with ordinary (non-weighted) LS obtained with Levenberg-Marquardt algorithm.

As it was mentioned in Section 2.3 it is necessary to remove the outliers before the AVAR estimation. For that purpose, the Hampel filter [40] was employed. It checks the signal whether it is larger than the 3 sigma rule threshold computed robustly on the median absolute deviation (MAD) within a sliding window (in our case 1 second of the past and future values) and replaces outlying values with the local median.

### 3.5. Remarks on the Results

During the data analysis, two issues emerged, it is worth mentioning them in advance as they could contaminate the results or cause confusion during the interpretation. First, that while recording there occurred a slight seismic crump. The second issue was the failure of one of the cameras (IR LED emitter) during the recording.

**Figure 7:**
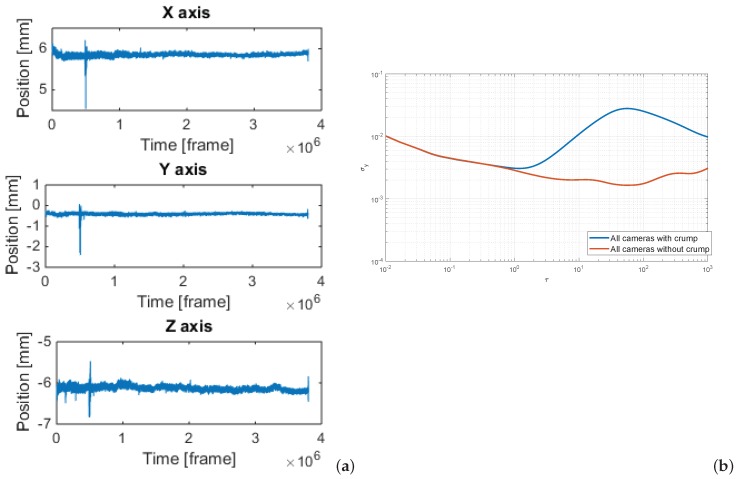
Position X,Y,Z of marker M1 based on data set 1 (**a**) with crump present, (**b**) ADEV of marker distance based on data with and without crump.

The crump can be observed in the trajectories of the markers (Figure 7a) as a heavy outlier. It has a significant effect on the Allan variance results (see Figure 7b). It is worth mentioning that the occurrence of seismic crump was not detected immediately. After the recording, none of the cameras’ sensors signaled a bump, which could indicate a system decalibration, as in the case of, for example, a direct impact on the scaffolding or a gunshot in the room, which we already tested in the lab. The crump itself was relatively small-similar disturbance would be probably caused by a person running next to markers. Moreover, the basic variance descriptors, which we checked for 5 min periods before and after crump do not indicate any change in the system performance. If the cameras were mounted on tripods, then such an event would probably have a much larger impact. However, that fact draws attention to the need for the careful screening of the measurements for the outliers and proper filtering if necessary–such as the aforementioned Hampel filter.

**Figure 8:**
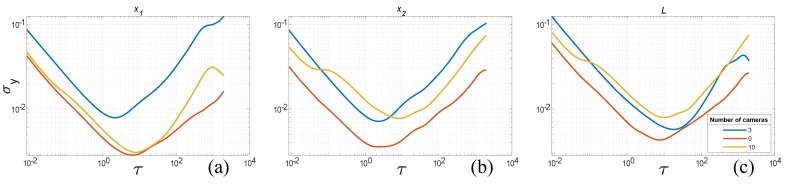
ADEV for Vantage cameras demonstrating the performance loss due to one damaged camera (tenth) for: (**a**) position *x*_1_, (**b**) position *x*_2_, (**c**) distance *L*

The second issue was identified because of the noise levels for data set 4 (Vantage cameras). They were aberrant, for some camera combination the noise levels were increasing when taking more cameras into the reconstruction process. It appeared that one of the cameras was out of order and it would have broken soon after our recordings. Therefore, we excluded it from our analyses and we used up to nine cameras in the reconstruction for the Vantage data set. Figure 8 illustrates how such a failing device, increases the noise in the results for *x* dimension–the most contributing to the length (*L*). It is visible in the figure that we observe larger ADEV values for 10 cameras than for 9, another noteworthy fact is that markers positions are affected to different extents, and the distance is therefore affected to an intermediate extent. The reason for such an observation remains unclear as the internal details of triangulation in Vicon software are kind of a black box. We suspect the inner quality control procedure, that could, for example, select just some subset of cameras.

There were also indirect consequences of the failing camera. We could observe slight cross-talk of distortions to the other cameras mounted at similar heights (Vantage and T40), resulting in the presence of short term correlated noise (verified in Section 4.6). For better or worse, it resulted finally in much more complex ADEV curves we had to analyze, proving that the proposed method is capable of adapting to all the noise types known from the Allan variance literature. The exemplary ADEV curve, obtained for the data after replacing the failing camera is included in Appendix B for comparison. It demonstrates ADEV of the whole system in the facility free of the distortions caused the failing IR LEDs.

This unexpected failure in the system is a premise for the conclusion, that statistical analysis of noise in the system could be a useful diagnostic procedure for early failure diagnostics as the LED flickering was imperceptible to the human observer, but was observable in the data. However, a profound analysis of errors and their sources is probably feasible at the manufacturer’s laboratory only. System vendor is probably the sole, who has knowledge of the appearing malfunctions, access to replaced devices such as ours, and who can induce misbehavior of the equipment on demand. Nevertheless, such a procedure, especially if coded into the system software, could become a part of system diagnostics and maintenance.

## 4. Results and Discussion

### 4.1. Overview

Using the single long sequence recorded with a regime as described in Section 3.1, Section 3.2 and Section 3.3, we have obtained a pool of sequences obtained with different camera sets. Initially (Section 4.2), we have analyzed the results using conventional Gaussian noise model as described in in Section 2.2. It appeared, as expected, to be insufficient to describe the noise present in the system. Therefore, the in-depth analysis was performed, and is described in successive steps.

The main analysis of results involved overlapping Allan variance estimator. First (Section 4.3) overall ADEV characteristics were obtained. These results are grouped by camera type and presented as families of Allan deviation plots in Figure 12 with a varying number of cameras used in the process.

Next, Allan variance noise component parameters were estimated with the procedure described in Section 3.4. The presence and number of correlated and periodic components were examined visually, the verification, whether they are not coming from the periodic distortion, was done by examination of the PSD estimator. The noise parameter estimation results are demonstrated in Figures 14 and 15, in logarithmic and linear scales to adapt to large range of values. Numerical values are attached in Appendix A in Table A1, Table A2 and Table A3.

Finally, the less conventional noises were considered in Section 4.6. These are multiple occurrences of correlated noise and periodic distortion.

### 4.2. Simple Gaussian Model

**Figure 9:**
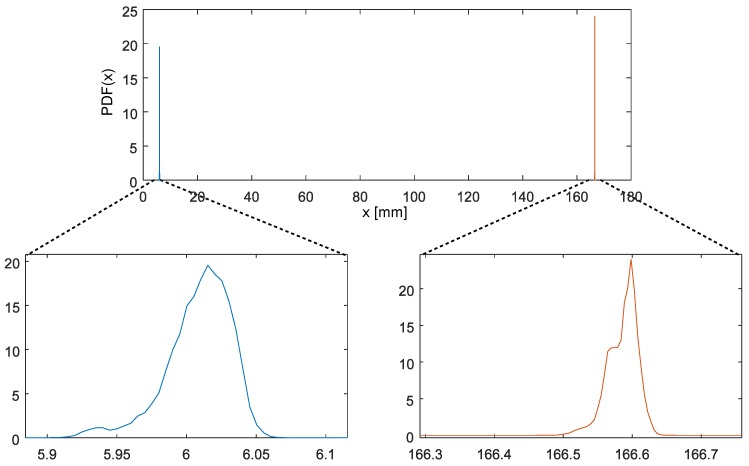
PDF kernel estimation of location for *M*_1_ and *M*_2_ using Vicon T40 cameras.

The brief results—markers location and their distance are gathered in Table 4. It contains estimated parameters for locations and lengths, we provide also theoretically calculated values for length. These are: locations mean values and their standard deviations, covariance, and correlation ($\rho_{12}$) as well, furthermore it contains mean value($\mu_{L}$) and standard deviation ($\sigma_{L}$) for length as it was reported by the Vicon software. Calculated statistical descriptors are the length ($\mu_{e}$) and standard deviation in four variants—$\sigma_{A..C}$ as listed in Section 2.2, with two *A* variants assuming either markers as a potential source of variance value. Figure 9 demonstrates exemplary kernel estimates of location PDFs for one of the camera sets.

Generally, the measurement results conform to the theoretic considerations for correlated random variables—obviously the length measurement results confirmed (not included in the paper) in Chi-squared statistical tests their origin in Gaussian distribution with $\sigma_{C}$. In the considered measurements it is visible in the dispersion of measurements, which is considered to be noise. For the low-cost Bonita cameras, the location variance is relatively large, moreover, it is non-correlated with each other (low correlation and covariance), therefore it can be considered to be noise. On the other hand, overall dispersion for the high-end T40 cameras is small but highly correlated. The high correlation coefficients mean that the measurement of precision with simple statistical descriptors needs to be extended in a more sophisticated way.

**Figure 10:**
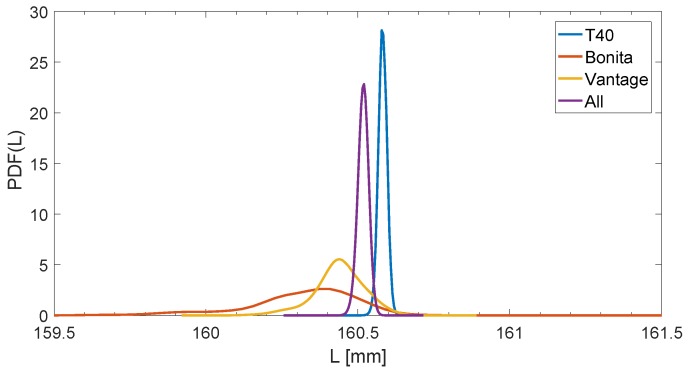
Variable PDF estimation of the same length measurement in OMC with different sets of cameras.

Concerning the precision, each of the camera sets reports slightly different mean value (see Figure 10), though the discrepancies between the camera sets are on the level rather satisfactory for the most applications: tenth part of a millimeter.

**Figure 11:**
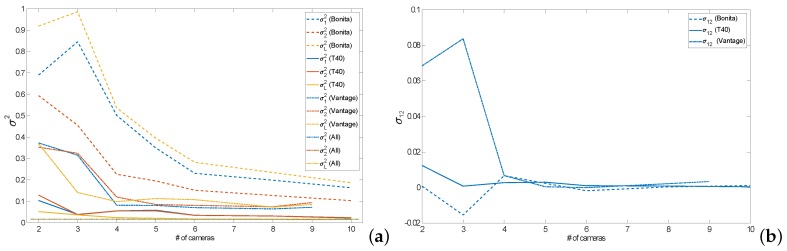
Variances (**a**) and covariances (**b**) for variable numbers of cameras of different types.

Regarding the number of cameras in the generic noise mode, the results are demonstrated in Figure 11, which demonstrates how the variances and covariances scales with increasing number of cameras used in the measurement. One can denote that the variance results adhere (with minor fluctuations) to the theoretical relationship given with Equation (3), it is clearly visible similarity between the theoretic characteristics in Figure 2 and the real observed decrease in variations for increasing number of cameras shown in Figure 11a.

Covariance, on the other hand, is rather constant regardless of the number of cameras (except very low numbers of cameras), as it is depicted in Figure 11b. It suggests the presence of a process of unknown origin that is rather common to the markers and affects their registration rather than physical devices, e.g., it could be either signal processing or a common mechanic micro trembling of cameras.

### 4.3. Overall ADEV Characteristics

**Figure 12:**
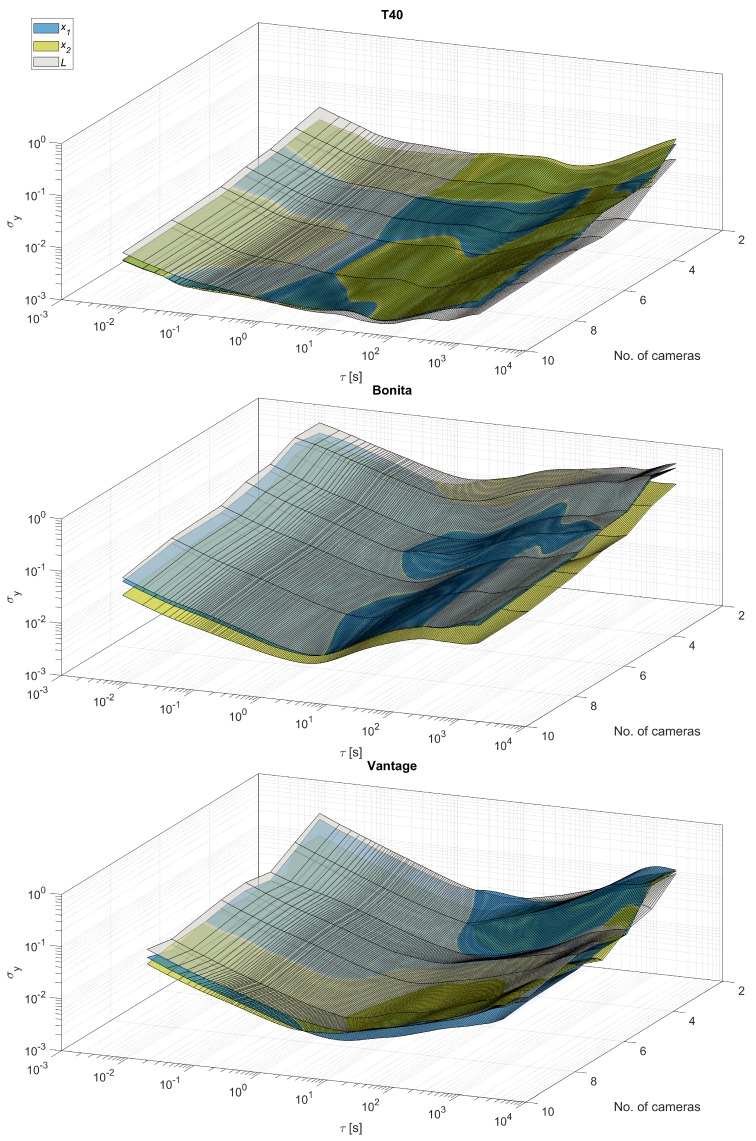
ADEV versus time plots for varying number of cameras (2...max) for three camera types, listed top–down: T40, Bonita, Vantage.

Overall ADEV characteristics for all camera types—T40, Bonita and Vantage—are shown in Figure 12. They reveal that the $\sigma_{y}$ values gradually decrease with the increasing number of cameras; however, there are some ’drops’ along the camera number axis. That suggests that certain camera setup is notably better suited for the recorded object. These setups are: 3 cameras for T40, 5 cameras for Bonita, and 4 cameras for Vantage.

Another interesting observation in the overall plots can be denoted when analyzing ADEV plots for measured values along the temporal axis. The $\sigma_{y}$ values for the distance *L* are larger than for the contributing locations $x_{1}$ and $x_{2}$ within the range of short-time noises $\tau \approx 10^{-2}\ldots 10^{1}$, although for the longer time ranges these values are on par or even ADEV values are smaller for the distance than for contributing locations. Apparently, flicker and random walk noises do not add in the system.

We can also observe all the slopes (−1, −1/2, 0, 1/2) from the Table 1 in the characteristics plots. The presence of random walk could be a bit confusing at first, if we omit the results of Jensenius [13], one could expect such distortion not to appear. The triangulation process is done frame-by-frame in the system, therefore the position of a marker should be steady, with flicker, white and higher frequency color noises present. However, there might be implicit denoising present in the system done by low pass filtering, such filters act as integrators, therefore they could introduce some low-frequency noises from the higher noise components.

### 4.4. Comparing the Camera Types

**Figure 13:**
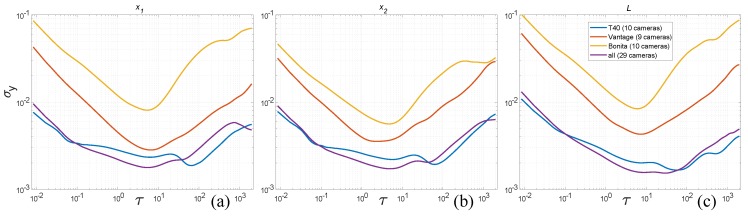
ADEV for maximal numbers of cameras of each type separately and altogether for: (**a**) position *x*_1_, (**b**) position *x*_2_, (**c**) distance *L*.

The logical corollary of results demonstrated in the previous paragraph is a direct comparison of camera types. In Figure 13 we demonstrate the ADEV plots for each of the camera types at its maximum performance configuration (all cameras). It shows length (*L*) and marker positions in *x* as the most important dimension. Comparing the camera types by visual plot inspection confirms the expected outcome, low-cost Bonita cameras have much higher ADEV values (are more noise affected) than two other camera types. However, ADEV values of up-to-date Vantage cameras indicate that they are more noise affected than relatively old T40s.

### 4.5. Estimation of Basic Noise Coefficients

**Figure 14:**
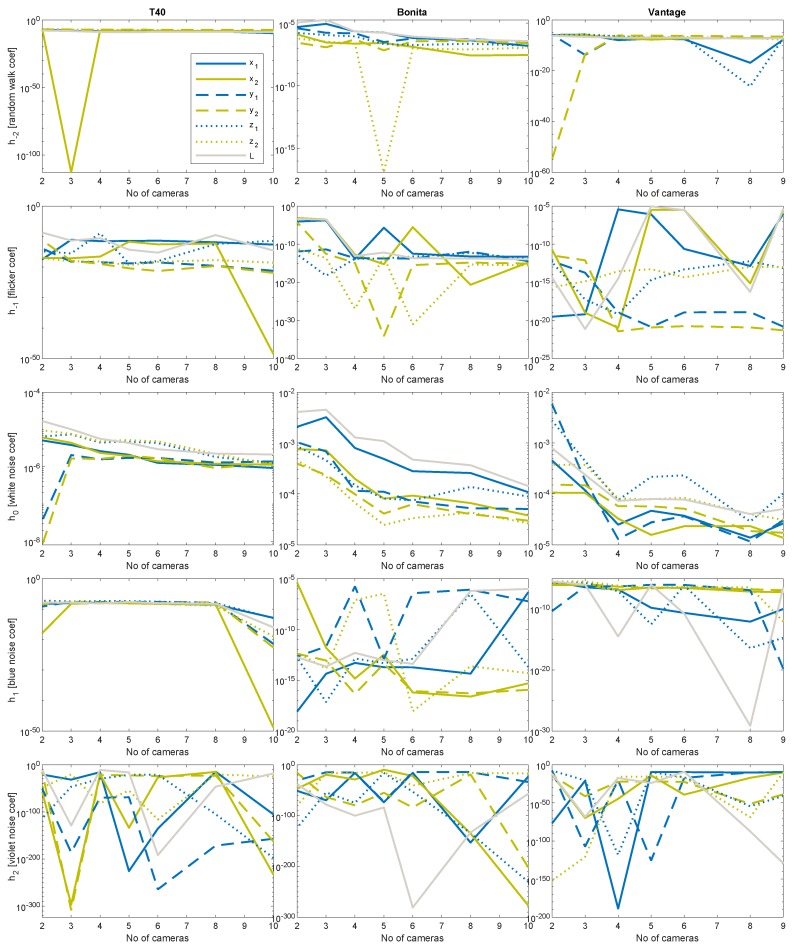
Color noises (*h*_−2_ … *h*_2_) coefficients in logarithmic scale; in columns left to right: T40, Bonita, Vantage.

**Figure 15:**
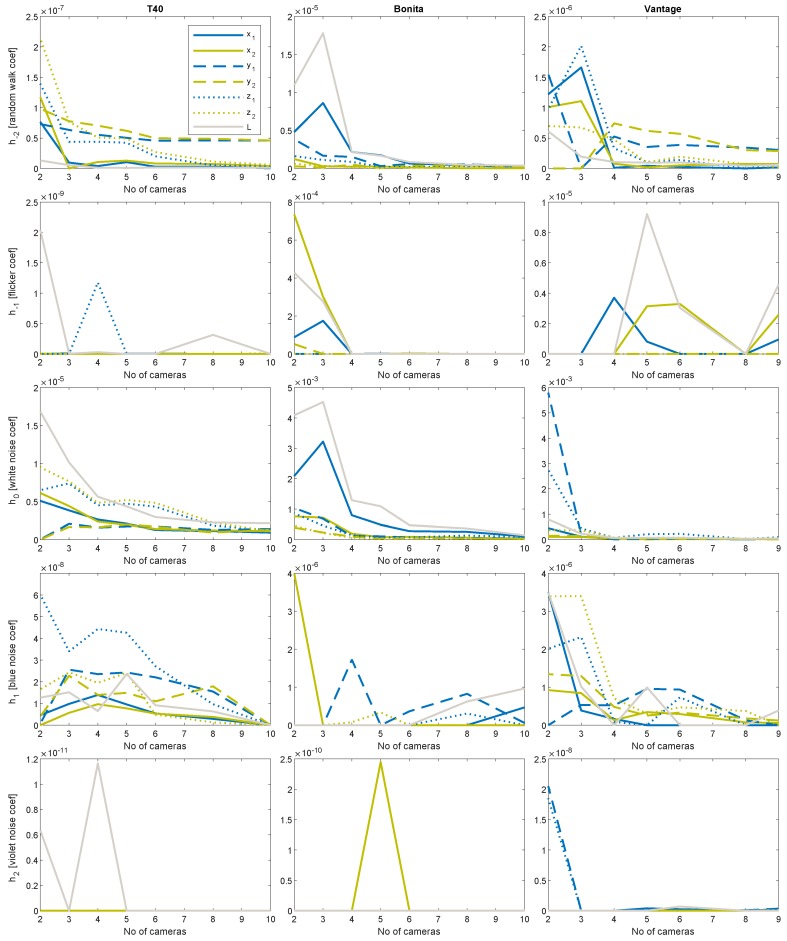
Color noises (*h*_−2_ … *h*_2_) coefficients in linear scale; in columns left to right: T40, Bonita, Vantage.

Several remarks on the noise colors present in the system that are based on the noise estimated coefficients (Figure 14 and Figure 15):

- Random walk and white noise diminishing with the increasing number of cameras, roughly follow quasi-hyperbolic characteristics described in Equation (3), $h\approx 10^{-5}$
- Flicker and blue noise levels orders of magnitude are relatively constant with low-moderate values $h\approx 10^{-10}$
- Violet noise levels are negligibly small $h\approx 10^{-20}\ldots 10^{-300}$

We could also observe intense peak fluctuations in plots of *h* coefficient characteristics. They could originate from two potential sources—numerical errors in the optimization process, and/or very specific camera geometric configuration—that could improve or degrade the results. However, the general rule (Equation (3)) of decreasing uncertainty with an increasing number of measurements could be observed to some extent for all the noise coefficients (*h*), but violet one, where it rather seems to be random fluctuations.

### 4.6. Unconventional Distortions

**Figure 16:**
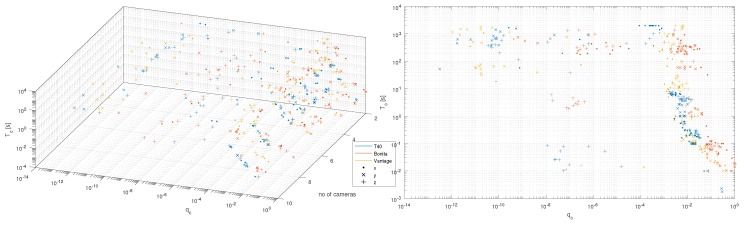
Distribution of correlated noises *q_c_* versus *T_c_*.

Regarding the less conventional, correlated noises, a series of interesting observations relates to their presence. In Figure 16a one can observe that they are mostly independent of the number of cameras; $T_{c}$ and $q_{c}$ parameters remain relatively constant. Moreover, one could note from Figure 16b that for correlated noises in the system the longer the time constant the lower amplitude. We could also identify three correlated noise ‘classes’ of a different correlation time constants. Their sources remain unknown; however, at least we could speculate about their origins based on $T_{c}$. Removing the failed camera made the first two of them (see Appendix B) disappear. Hence our guesses are:

- $10^{-2}$–$10^{0}$ s—due to failed camera, though we considered signal processing-based first,
- $10^{0}$–$10^{1}$ s—due to failed camera, at first we suspected mechanical-based microtrembling of camera support,
- $10^{2}$–$10^{3}$ s—environmental-based such as changes in room temperature.

**Figure 17:**
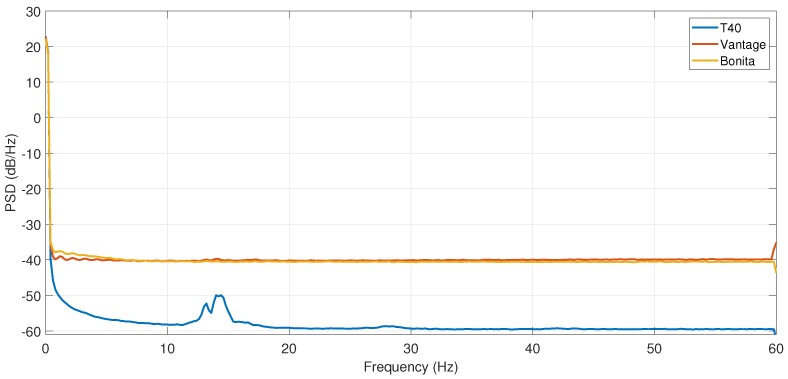
Exemplary verification of periodic distortion using PSD for *z*_1_ reconstructed with maximal number of devices for each camera type.

Finally, the occurrences of periodic noise of $f_{0}\approx 15$ Hz frequency had to be checked as it was an unexpected outcome. The verification was done using Welch estimator of PSD (see Figure 17), and it is present in each of the reconstructions using T40 and Vantage cameras. However, it is usually negligibly small phenomenon, barely observable in most of ADEV plots. Its origin cannot be connected with a recording of any specific camera, but since all the cameras were recording concurrently, it is probably due to some environmental source. Cameras appeared sensitive to a different extent, surprisingly vertical dimensions obtained from the T40 cameras were the most sensitive to this distortion. In case of the Vantage or Bonita cameras it is negligibly small. It requires very large zoom to observe appearance of respective bumps in either PSD or ADEV plots.

## 5. Summary

In the article, we demonstrated how to evaluate with Allan variance a compound structure of noise present in the optical Mocap system. The proposed tool was invented for situations when the reliable ground truth is inaccessible. We demonstrated that it provides outcomes convenient for visual inspection to identify qualitatively noise types actually present in the system or to compare two systems. We have also demonstrated how to employ sophisticated solvers to read the noise parameters from the characteristic ADEV curve, even when it gets quite complicated form.

For our facility, we proved that the main contribution to the imprecision comes from the random walk and white noise with coefficients being $h\approx 10^{-5}$, whereas flicker noise and blue noise contributions are several orders of magnitude smaller of a $h\approx 10^{-10}$. The influence of violet noise is negligibly small $h\approx 10^{-300}\ldots 10^{-20}$. We have identified also the presence of quite a long term (tens of minutes) correlated noise, probably due to environmental influence and periodic distortion with time constant of a minute order($T_{c}\approx 10^{2}\ldots 10^{3}$) and notable amplitude amplitude ($q_{c}=10^{-3}\ldots 10^{-13}$). Additionally, we identified a periodic distortion of 15 Hz frequency, which is visible to the cameras reversely to their overall quality, with an amplitude of $A_{s}\approx 10^{-3}$.

The registration of the noise connected with camera failure was an additional and unforeseen outcome; however this might be seminal for establishing statistical based diagnostics procedure for the motion capture laboratories.

It should be noted that the conditions during the experiment significantly differed from the conditions during the traditional MoCap session. The noise resulting from the behavior of actors (running, jumping, screaming, etc.), as well as people responsible for the session (technical staff, art director, etc.), are much greater than those recorded in ideal conditions. The data during the post-processing stage are also filtered, using Butterworth or Woltring filter. Of course, some attempts can be made to reduce the impact of certain types of noses.

Some recommendations can be made for noise suppression in the OMC system. Part of the observed distortion is very simple to suppress with low pass filtering: white noise and above noises are very convenient cases. Also, periodic noise can be easily addressed with band-stop filters, at least as long we are aware of its presence and its frequency is not too low. However, longer-term distortions, such as flicker or random walk are quite inconvenient to remove with simple filters, but the multiresolution approach seems to be promising to identify and suppress noises of longer duration than single samples. Finally, regarding the environmental-based distortion, modifying overall lab conditions could help to reduce such noises, such modifications as employing continuous air-conditioning, low heat light sources, door insulation, and the like are worth further testing.

Future works might include extending the basic approach by analysis of AVAR for bone orientation, though it would require preparing quaternionic AVAR. Other interesting aspects are the variability of AVAR depending on the location in the scene, different marker sizes, or how much the results are affected by the presence and activities of staff in the lab. Prospective dynamic tests are possible to some extent only, since AVAR is based on assumption of static signal, because computing of AVAR is not possible for moving markers. However, if we assume T-frame or any other template is stiff enough to be considered to be a rigid body, then we can consider computing AVAR for the inter-marker distance regardless it is steady or in motion. The experiments could be also repeated in different Mocap facilities or laboratories of a different kind, but including Mocap as a feature such as interactive rehabilitation platform Motek CAREN (The Computer Assisted Rehabilitation ENvironment)—https://www.motekmedical.com/product/caren/.

## Figures and Tables

**Table 1:** Power-law noise types and their Allan variance representation [35].

Noise Name	*α*	*μ*	*K_α_*	*σ*^2^(*τ*)
Random walk	−2	1	$A=\frac{2\pi^{2}}{3}$	$\sigma_{r}^{2}\left(\tau \right)=\frac{2\pi^{2}}{3}h_{-2}\tau$
Flicker (pink) noise	−1	0	B=2ln2	$\sigma_{f}^{2}\left(\tau \right)=2\ln\left(2\right)h_{-1}$
White noise	0	−1	$C=\frac{1}{2}$	$\sigma_{w}^{2}\left(\tau \right)=\frac{1}{2}h_{0}\tau^{-1}$
Blue	1	−2	$D=\frac{1.038+3\ln w_{h}\tau }{4\pi^{2}}$	$\sigma_{b}^{2}\left(\tau \right)=\frac{1.038+3\ln2\pi f_{h}\tau }{4\pi^{2}}h_{1}\tau^{-2}$
Violet	2	−2	$E=\frac{3f_{h}}{4\pi^{2}}$	$\sigma_{v}^{2}\left(\tau \right)=\frac{3f_{h}}{4\pi^{2}}h_{2}\tau^{-2}$

**Table 2:** Vicon camera difference.

Camera Model	MX-T40	Bonita10	Vantage V5
Resolution [MP]	4	1	5
Max Frame Rate [HZ]	370 @ 4 MP	250 @ 1 MP	420 @ 5 MP
Focal length [mm]	18	4	8.5
Sensor type	CMOS	CMOSIS	CMOS
Type of, LEDs	180 nm NIR	780 nm NIR	850 nm (IR)
Number of LEDs	252	68	22
AOV (H × V)	49.15 × 37.14	70.29 × 70.29	63.5 × 55.1
Dimensions [mm], (H × W × D)	207 × 130 × 75	122 × 80 × 79	166.2 × 125 × 134.1
Weight [kg]	1.8	1	1.6

**Table 3:** Number and (incrementally) IDs of cameras used for 3D marker reconstruction.

Cameras	MX-T40	Bonita10	Vantage V5
2	21.26	2.17	3.4
3	+28	+19	+10
4	+22	+16	+11
5	+27	+18	+7
6	+20	+15	+30
8	+24; 29	+13; 14	+9; 8
ALL	+23; 25	+1; 12	+5

**Table 4:** Stats.

	Measured [mm]								Theoretic [mm]				
	*μ*_1_	*σ*_1_	*μ*_2_	*σ*_2_	*σ*_12_	*ρ*_12_	*μ_L_*	*σ_L_*	*μ_e_*	*σ_A_*_1_	*σ_A_*_2_	*σ_B_*	*σ_C_*
**T40**	166.5863	0.0217	6.0088	0.0234	0.0004	0.8006	160.5818	0.0143	160.5774	0.0307	0.0331	0.0319	0.0143
**Bonita**	165.9766	0.1635	5.6644	0.1032	0.0011	0.0670	160.3168	0.1870	160.3121	0.2312	0.1459	0.1933	0.1874
**Vantage**	166.1736	0.0721	5.7363	0.0942	0.0034	0.4980	160.4388	0.0852	160.4374	0.1020	0.1332	0.1186	0.0855
**All**	166.4613	0.0157	5.9478	0.0176	0.0001	0.4257	160.5176	0.0178	160.5136	0.0222	0.0249	0.0236	0.0179

**Figure A1:**
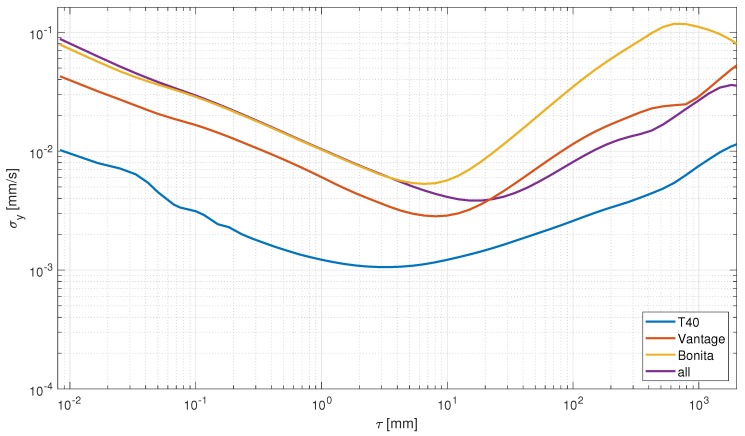
Exemplary Allan deviation for supplementary recording—*z*_1_.

## Abbreviations

The following abbreviations are used in this manuscript:

ACO	ant colony optimization
AVAR	Allan variance
ADEV	Allan deviation
LS	least squares
Mocap	motion capture
OMC	optical motion capture
RMSE	root mean square error
PSD	power spectral density
SA	simulated annealing
SD	standard deviation
WLS	weighted least squares

## Appendix A. Estimated Noise Coefficients

Allan variance numerical values for noise component parameters, estimated with the procedure described in Section 3.4. Table A1, Table A2 and Table A3 demonstrate results for T40, Bonita, and Vantage cameras respectively.

Table A1 Estimated noise parameters of T40 cameras type for 2..10 devices.

**Table:** (a) x_1_

	*h*_−2_	*h*_−1_	*h*_0_	*h*_1_	*h*_2_	*q_c_*_1_	*T_c_*_1_	*q_c_*_2_	*T_c_*_2_	*q_c_*_3_	*T*_3_	*q_c_*_4_	*T_c_*_4_
**All**	$1.55\times 10^{-10}$	$2.37\times 10^{-13}$	$9.26\times 10^{-07}$	$1.35\times 10^{-13}$	$5.59\times 10^{-106}$	$5.00\times 10^{-02}$	$1.00\times 10^{-02}$	$1.33\times 10^{-02}$	$2.25\times 10^{-01}$	$2.04\times 10^{-03}$	$5.859\times 10^{+00}$	$3.60\times 10^{-04}$	$2.00\times 10^{+03}$
**8**	$2.99\times 10^{-09}$	$1.33\times 10^{-12}$	$1.12\times 10^{-06}$	$2.91\times 10^{-09}$	$2.01\times 10^{-15}$	$5.56\times 10^{-03}$	$1.62\times 10^{-01}$	$9.80\times 10^{-03}$	$3.00\times 10^{-01}$	$1.92\times 10^{-03}$	$6.36\times 10^{+00}$	$2.39\times 10^{-04}$	$2.00\times 10^{+03}$
**6**	$2.82\times 10^{-09}$	$4.50\times 10^{-12}$	$1.27\times 10^{-06}$	$5.42\times 10^{-09}$	$4.54\times 10^{-136}$	$4.93\times 10^{-03}$	$5.48\times 10^{-01}$	$1.21\times 10^{-02}$	$2.05\times 10^{-01}$	$2.13\times 10^{-03}$	$7.03\times 10^{+00}$	$3.20\times 10^{-04}$	$2.00\times 10^{+03}$
**5**	$1.03\times 10^{-08}$	$3.62\times 10^{-12}$	$2.09\times 10^{-06}$	$9.57\times 10^{-09}$	$3.92\times 10^{-226}$	$5.38\times 10^{-03}$	$5.63\times 10^{-01}$	$1.43\times 10^{-02}$	$2.42\times 10^{-01}$	$3.20\times 10^{-03}$	$6.30\times 10^{+00}$	$3.66\times 10^{-04}$	$2.00\times 10^{+03}$
**4**	$3.96\times 10^{-09}$	$3.24\times 10^{-12}$	$2.63\times 10^{-06}$	$1.39\times 10^{-08}$	$2.40\times 10^{-16}$	$1.99\times 10^{-02}$	$1.99\times 10^{-01}$	$7.45\times 10^{-03}$	$6.35\times 10^{-01}$	$3.12\times 10^{-03}$	$7.22\times 10^{+00}$	$6.01\times 10^{-04}$	$2.00\times 10^{+03}$
**3**	$9.57\times 10^{-09}$	$9.04\times 10^{-12}$	$3.83\times 10^{-06}$	$9.82\times 10^{-09}$	$3.15\times 10^{-32}$	$2.09\times 10^{-02}$	$1.95\times 10^{-01}$	$5.62\times 10^{-03}$	$5.68\times 10^{-01}$	$4.60\times 10^{-03}$	$6.57\times 10^{+00}$	$6.51\times 10^{-04}$	$1.41\times 10^{+03}$
**2**	$7.65\times 10^{-08}$	$3.26\times 10^{-18}$	$5.09\times 10^{-06}$	$4.60\times 10^{-09}$	$1.96\times 10^{-21}$	$9.28\times 10^{-03}$	$4.54\times 10^{+00}$	$2.46\times 10^{-02}$	$1.79\times 10^{-01}$	$7.63\times 10^{-10}$	$5.74\times 10^{+01}$	$5.36\times 10^{-10}$	$1.55\times 10^{+03}$

**Table:** (b) *y*_1_

	*h*_−2_	*h*_−1_	*h*_0_	*h*_1_	*h*_2_	*q_c_*_1_	*T_c_*_1_	*q_c_*_2_	*T_c_*_2_	*q_c_*_3_	*T_c_*_3_	*As*	*f*_0_
**All**	$4.59\times 10^{-08}$	$6.11\times 10^{-22}$	$1.40\times 10^{-06}$	$3.91\times 10^{-22}$	$1.47\times 10^{-157}$	$1.58\times 10^{-02}$	$1.00\times 10^{-01}$	$4.72\times 10^{-03}$	$1.00\times 10^{+00}$	$6.36\times 10^{-12}$	$6.21\times 10^{+02}$	$1.75\times 10^{-03}$	$5.00\times 10^{-02}$
**8**	$4.62\times 10^{-08}$	$2.45\times 10^{-20}$	$1.29\times 10^{-06}$	$1.55\times 10^{-08}$	$3.73\times 10^{-172}$	$1.41\times 10^{-02}$	$1.00\times 10^{-01}$	$3.78\times 10^{-03}$	$1.93\times 10^{+00}$	$6.78\times 10^{-11}$	$6.73\times 10^{+02}$	$1.22\times 10^{-03}$	$2.98\times 10^{-01}$
**6**	$4.56\times 10^{-08}$	$3.32\times 10^{-19}$	$1.72\times 10^{-06}$	$2.21\times 10^{-08}$	$6.14\times 10^{-265}$	$1.71\times 10^{-02}$	$1.00\times 10^{-01}$	$4.26\times 10^{-03}$	$2.72\times 10^{+00}$	$4.65\times 10^{-11}$	$3.65\times 10^{+02}$	$1.75\times 10^{-03}$	$2.98\times 10^{-01}$
**5**	$5.04\times 10^{-08}$	$1.59\times 10^{-19}$	$1.73\times 10^{-06}$	$2.43\times 10^{-08}$	$1.50\times 10^{-69}$	$1.76\times 10^{-02}$	$1.00\times 10^{-01}$	$4.62\times 10^{-03}$	$2.70\times 10^{+00}$	$5.84\times 10^{-11}$	$8.47\times 10^{+02}$	$1.72\times 10^{-03}$	$2.98\times 10^{-01}$
**4**	$5.53\times 10^{-08}$	$3.88\times 10^{-19}$	$1.58\times 10^{-06}$	$2.35\times 10^{-08}$	$2.12\times 10^{-70}$	$1.99\times 10^{-02}$	$1.00\times 10^{-01}$	$5.84\times 10^{-03}$	$2.54\times 10^{+00}$	$8.04\times 10^{-11}$	$6.60\times 10^{+02}$	$1.51\times 10^{-03}$	$2.98\times 10^{-01}$
**3**	$6.34\times 10^{-08}$	$5.93\times 10^{-19}$	$2.06\times 10^{-06}$	$2.56\times 10^{-08}$	$8.38\times 10^{-187}$	$2.03\times 10^{-02}$	$1.00\times 10^{-01}$	$5.74\times 10^{-03}$	$3.62\times 10^{+00}$	$9.94\times 10^{-11}$	$3.88\times 10^{+02}$	$1.38\times 10^{-03}$	$2.98\times 10^{-01}$
**2**	$7.30\times 10^{-08}$	$1.29\times 10^{-14}$	$3.58\times 10^{-08}$	$7.00\times 10^{-10}$	$1.29\times 10^{-51}$	$2.91\times 10^{-01}$	$1.76\times 10^{-03}$	$8.38\times 10^{-03}$	$3.72\times 10^{+00}$	$2.22\times 10^{-08}$	$9.20\times 10^{+02}$	$2.99\times 10^{-03}$	$5.00\times 10^{-02}$

**Table:** (c) *z*_1_

	*h*_−2_	*h*_−1_	*h*_0_	*h*_1_	*h*_2_	*q_c_*_1_	*T_c_*_1_	*q_c_*_2_	*T_c_*_2_	*q_c_*_3_	*T_c_*_3_	*q_c_*_4_	*T_c_*_4_	*As*	*f*_0_
**All**	$3.23\times 10^{-09}$	$4.20\times 10^{-12}$	$1.26\times 10^{-06}$	$1.04\times 10^{-13}$	$3.12\times 10^{-198}$	$1.87\times 10^{-02}$	$1.00\times 10^{-01}$	$6.76\times 10^{-03}$	$5.10\times 10^{-01}$	$1.69\times 10^{-03}$	$7.75\times 10^{+00}$	$1.70\times 10^{-04}$	$4.43\times 10^{+02}$	$3.56\times 10^{-03}$	$1.44\times 10^{+01}$
**8**	$5.04\times 10^{-09}$	$3.53\times 10^{-13}$	$1.83\times 10^{-06}$	$9.68\times 10^{-09}$	$1.36\times 10^{-105}$	$3.15\times 10^{-06}$	$5.29\times 10^{-02}$	$1.23\times 10^{-02}$	$3.22\times 10^{-01}$	$2.29\times 10^{-03}$	$7.66\times 10^{+00}$	$2.48\times 10^{-04}$	$6.75\times 10^{+02}$	$4.19\times 10^{-03}$	$1.44\times 10^{+01}$
**6**	$2.00\times 10^{-08}$	$8.84\times 10^{-19}$	$4.33\times 10^{-06}$	$2.72\times 10^{-08}$	$1.16\times 10^{-21}$	$5.48\times 10^{-08}$	$7.75\times 10^{-02}$	$2.11\times 10^{-02}$	$2.95\times 10^{-01}$	$4.17\times 10^{-03}$	$5.20\times 10^{+00}$	$7.09\times 10^{-11}$	$3.98\times 10^{+02}$	$6.69\times 10^{-03}$	$1.44\times 10^{+01}$
**5**	$4.22\times 10^{-08}$	$1.61\times 10^{-19}$	$4.71\times 10^{-06}$	$4.26\times 10^{-08}$	$4.48\times 10^{-22}$	$2.31\times 10^{-08}$	$2.61\times 10^{-02}$	$2.32\times 10^{-02}$	$2.74\times 10^{-01}$	$5.26\times 10^{-03}$	$4.24\times 10^{+00}$	$7.81\times 10^{-11}$	$7.27\times 10^{+02}$	$5.30\times 10^{-03}$	$1.44\times 10^{+01}$
**4**	$4.40\times 10^{-08}$	$1.18\times 10^{-09}$	$4.49\times 10^{-06}$	$4.44\times 10^{-08}$	$2.16\times 10^{-30}$	$2.06\times 10^{-02}$	$9.77\times 10^{-02}$	$2.30\times 10^{-02}$	$3.06\times 10^{-01}$	$5.31\times 10^{-03}$	$4.38\times 10^{+00}$	$6.32\times 10^{-07}$	$4.34\times 10^{+02}$	$1.69\times 10^{-01}$	$2.21\times 10^{+04}$
**3**	$4.39\times 10^{-08}$	$3.06\times 10^{-16}$	$7.38\times 10^{-06}$	$3.40\times 10^{-08}$	$2.34\times 10^{-47}$	$1.57\times 10^{-05}$	$1.44\times 10^{-02}$	$2.91\times 10^{-02}$	$2.19\times 10^{-01}$	$6.44\times 10^{-03}$	$5.22\times 10^{+00}$	$3.55\times 10^{-04}$	$1.00\times 10^{+03}$	$4.31\times 10^{-01}$	$1.03\times 10^{+18}$
**2**	$1.39\times 10^{-07}$	$1.44\times 10^{-15}$	$6.49\times 10^{-06}$	$6.02\times 10^{-08}$	$2.54\times 10^{-114}$	$6.43\times 10^{-07}$	$8.02\times 10^{-02}$	$3.32\times 10^{-02}$	$1.53\times 10^{-01}$	$1.33\times 10^{-02}$	$3.87\times 10^{+00}$	$1.19\times 10^{-08}$	$5.01\times 10^{+02}$	$6.25\times 10^{-03}$	$1.41\times 10^{+01}$

**Table:** (d) *x*_2_

	*h*_−2_	*h*_−1_	*h*_0_	*h*_1_	*h*_2_	*q_c_*_1_	*T_c_*_1_	*q_c_*_2_	*T_c_*_2_	*q_c_*_3_	*T*_3_	*q_c_*_4_	*T_c_*_4_
**All**	$3.87\times 10^{-09}$	$2.76\times 10^{-49}$	$1.13\times 10^{-06}$	$1.48\times 10^{-49}$	$1.15\times 10^{-233}$	$1.16\times 10^{-02}$	$1.20\times 10^{-01}$	$7.82\times 10^{-03}$	$3.20\times 10^{-01}$	$1.84\times 10^{-03}$	$6.67\times 10^{+00}$	$1.58\times 10^{-04}$	$2.00\times 10^{+03}$
**8**	$7.28\times 10^{-09}$	$5.06\times 10^{-13}$	$1.21\times 10^{-06}$	$3.81\times 10^{-09}$	$7.99\times 10^{-16}$	$8.67\times 10^{-03}$	$2.00\times 10^{-01}$	$7.56\times 10^{-03}$	$3.00\times 10^{-01}$	$2.11\times 10^{-03}$	$5.85\times 10^{+00}$	$2.68\times 10^{-07}$	$6.23\times 10^{+02}$
**6**	$8.11\times 10^{-09}$	$4.65\times 10^{-12}$	$1.40\times 10^{-06}$	$5.46\times 10^{-09}$	$1.46\times 10^{-14}$	$1.17\times 10^{-02}$	$1.95\times 10^{-01}$	$6.44\times 10^{-03}$	$3.68\times 10^{-01}$	$2.34\times 10^{-03}$	$6.27\times 10^{+00}$	$8.61\times 10^{-05}$	$2.00\times 10^{+03}$
**5**	$1.28\times 10^{-08}$	$6.62\times 10^{-13}$	$1.89\times 10^{-06}$	$9.90\times 10^{-09}$	$7.36\times 10^{-175}$	$1.36\times 10^{-02}$	$2.00\times 10^{-01}$	$7.82\times 10^{-03}$	$4.21\times 10^{-01}$	$3.06\times 10^{-03}$	$5.69\times 10^{+00}$	$2.65\times 10^{-04}$	$2.00\times 10^{+03}$
**4**	$1.06\times 10^{-08}$	$7.01\times 10^{-13}$	$2.36\times 10^{-06}$	$9.55\times 10^{-09}$	$6.19\times 10^{-43}$	$2.02\times 10^{-02}$	$1.94\times 10^{-01}$	$7.42\times 10^{-03}$	$5.61\times 10^{-01}$	$3.27\times 10^{-03}$	$6.42\times 10^{+00}$	$4.45\times 10^{-04}$	$2.00\times 10^{+03}$
**3**	$5.84\times 10^{-14}$	$1.90\times 10^{-13}$	$4.40\times 10^{-06}$	$5.61\times 10^{-09}$	$1.75\times 10^{-50}$	$2.38\times 10^{-02}$	$1.79\times 10^{-01}$	$7.00\times 10^{-03}$	$6.40\times 10^{-01}$	$4.99\times 10^{-03}$	$6.26\times 10^{+00}$	$7.95\times 10^{-04}$	$1.59\times 10^{+03}$
**2**	$1.17\times 10^{-07}$	$6.66\times 10^{-18}$	$6.11\times 10^{-06}$	$2.10\times 10^{-17}$	$1.25\times 10^{-33}$	$2.83\times 10^{-02}$	$1.85\times 10^{-01}$	$2.75\times 10^{-03}$	$8.07\times 10^{-01}$	$1.12\times 10^{-02}$	$4.24\times 10^{+00}$	$1.54\times 10^{-10}$	$1.25\times 10^{+03}$

**Table:** (e) *y*_2_

	*h*_−2_	*h*_−1_	*h*_0_	*h*_1_	*h*_2_	*q_c_*_1_	*T_c_*_1_	*q_c_*_2_	*T_c_*_2_	*q_c_*_3_	*T_c_*_3_	*As*	*f*_0_
**All**	$4.60\times 10^{-08}$	$1.19\times 10^{-22}$	$1.29\times 10^{-06}$	$2.56\times 10^{-23}$	$1.83\times 10^{-163}$	$1.55\times 10^{-02}$	$1.00\times 10^{-01}$	$4.85\times 10^{-03}$	$1.00\times 10^{+00}$	$1.78\times 10^{-12}$	$6.32\times 10^{+02}$	$1.34\times 10^{-03}$	$3.69\times 10^{-02}$
**8**	$4.87\times 10^{-08}$	$2.22\times 10^{-20}$	$9.29\times 10^{-07}$	$1.79\times 10^{-08}$	$3.87\times 10^{-23}$	$1.55\times 10^{-02}$	$1.00\times 10^{-01}$	$4.86\times 10^{-03}$	$1.37\times 10^{+00}$	$3.11\times 10^{-13}$	$5.27\times 10^{+01}$	$3.82\times 10^{-03}$	$1.37\times 10^{+01}$
**6**	$4.97\times 10^{-08}$	$4.97\times 10^{-22}$	$1.69\times 10^{-06}$	$1.10\times 10^{-08}$	$9.16\times 10^{-25}$	$1.68\times 10^{-02}$	$1.00\times 10^{-01}$	$6.71\times 10^{-03}$	$1.00\times 10^{+00}$	$1.71\times 10^{-12}$	$4.53\times 10^{+02}$	$2.51\times 10^{-03}$	$2.68\times 10^{-02}$
**5**	$6.18\times 10^{-08}$	$3.91\times 10^{-21}$	$1.99\times 10^{-06}$	$1.49\times 10^{-08}$	$4.88\times 10^{-23}$	$1.76\times 10^{-02}$	$1.00\times 10^{-01}$	$7.23\times 10^{-03}$	$1.03\times 10^{+00}$	$7.33\times 10^{-11}$	$1.42\times 10^{+03}$	$3.50\times 10^{-03}$	$2.35\times 10^{-02}$
**4**	$7.04\times 10^{-08}$	$1.11\times 10^{-19}$	$1.62\times 10^{-06}$	$1.40\times 10^{-08}$	$8.68\times 10^{-23}$	$2.07\times 10^{-02}$	$1.00\times 10^{-01}$	$7.57\times 10^{-03}$	$1.45\times 10^{+00}$	$5.02\times 10^{-11}$	$5.73\times 10^{+02}$	$3.77\times 10^{-03}$	$2.40\times 10^{-02}$
**3**	$7.77\times 10^{-08}$	$1.15\times 10^{-18}$	$1.67\times 10^{-06}$	$2.28\times 10^{-08}$	$2.64\times 10^{-309}$	$2.12\times 10^{-02}$	$1.00\times 10^{-01}$	$7.22\times 10^{-03}$	$3.65\times 10^{+00}$	$1.15\times 10^{-10}$	$1.20\times 10^{+03}$	$3.66\times 10^{-03}$	$1.41\times 10^{+01}$
**2**	$9.83\times 10^{-08}$	$5.57\times 10^{-12}$	$8.11\times 10^{-09}$	$3.93\times 10^{-09}$	$1.25\times 10^{-15}$	$2.75\times 10^{-01}$	$2.30\times 10^{-03}$	$9.90\times 10^{-03}$	$2.74\times 10^{+00}$	$6.15\times 10^{-07}$	$9.29\times 10^{+02}$	$6.39\times 10^{-03}$	$1.95\times 10^{-02}$

**Table:** (f) *z*_2_

	*h*_−2_	*h*_−1_	*h*_0_	*h*_1_	*h*_2_	*q_c_*_1_	*T_c_*_1_	*q_c_*_2_	*T_c_*_2_	*q_c_*_3_	*T_c_*_3_	*q_c_*_4_	*T_c_*_4_	*As*	*f*_0_
**All**	$6.01\times 10^{-09}$	$2.51\times 10^{-19}$	$1.11\times 10^{-06}$	$1.10\times 10^{-19}$	$1.56\times 10^{-25}$	$7.49\times 10^{-02}$	$1.00\times 10^{-02}$	$1.56\times 10^{-02}$	$1.98\times 10^{-01}$	$2.20\times 10^{-03}$	$5.61\times 10^{+00}$	$5.15\times 10^{-11}$	$4.07\times 10^{+02}$	$1.08\times 10^{-03}$	$1.70\times 10^{-01}$
**8**	$1.13\times 10^{-08}$	$1.98\times 10^{-18}$	$2.22\times 10^{-06}$	$1.29\times 10^{-09}$	$2.76\times 10^{-21}$	$5.57\times 10^{-08}$	$1.03\times 10^{-02}$	$1.31\times 10^{-02}$	$2.67\times 10^{-01}$	$2.63\times 10^{-03}$	$6.34\times 10^{+00}$	$1.09\times 10^{-10}$	$2.01\times 10^{+02}$	$1.11\times 10^{-03}$	$1.81\times 10^{-01}$
**6**	$2.68\times 10^{-08}$	$5.11\times 10^{-19}$	$4.83\times 10^{-06}$	$4.89\times 10^{-09}$	$6.68\times 10^{-117}$	$2.57\times 10^{-08}$	$2.65\times 10^{-02}$	$2.16\times 10^{-02}$	$2.52\times 10^{-01}$	$4.65\times 10^{-03}$	$3.95\times 10^{+00}$	$5.00\times 10^{-11}$	$3.20\times 10^{+02}$	$1.50\times 10^{-03}$	$1.92\times 10^{-01}$
**5**	$4.92\times 10^{-08}$	$3.92\times 10^{-20}$	$5.20\times 10^{-06}$	$2.41\times 10^{-08}$	$2.20\times 10^{-55}$	$3.60\times 10^{-08}$	$2.53\times 10^{-02}$	$2.26\times 10^{-02}$	$2.38\times 10^{-01}$	$5.64\times 10^{-03}$	$4.02\times 10^{+00}$	$3.51\times 10^{-11}$	$3.69\times 10^{+02}$	$2.01\times 10^{-03}$	$2.63\times 10^{-01}$
**4**	$5.09\times 10^{-08}$	$7.10\times 10^{-19}$	$4.83\times 10^{-06}$	$1.95\times 10^{-08}$	$1.63\times 10^{-82}$	$9.90\times 10^{-08}$	$1.67\times 10^{-02}$	$2.76\times 10^{-02}$	$2.21\times 10^{-01}$	$5.93\times 10^{-03}$	$3.81\times 10^{+00}$	$1.57\times 10^{-10}$	$8.56\times 10^{+02}$	$1.93\times 10^{-03}$	$2.17\times 10^{-01}$
**3**	$7.67\times 10^{-08}$	$7.72\times 10^{-19}$	$7.65\times 10^{-06}$	$2.43\times 10^{-08}$	$4.60\times 10^{-21}$	$1.12\times 10^{-08}$	$8.42\times 10^{-02}$	$3.15\times 10^{-02}$	$1.71\times 10^{-01}$	$7.61\times 10^{-03}$	$4.47\times 10^{+00}$	$1.30\times 10^{-10}$	$5.86\times 10^{+02}$	$2.28\times 10^{-03}$	$3.83\times 10^{-01}$
**2**	$2.14\times 10^{-07}$	$8.77\times 10^{-18}$	$9.50\times 10^{-06}$	$1.65\times 10^{-08}$	$8.94\times 10^{-49}$	$3.99\times 10^{-08}$	$5.36\times 10^{-02}$	$3.34\times 10^{-02}$	$1.65\times 10^{-01}$	$1.52\times 10^{-02}$	$3.96\times 10^{+00}$	$6.98\times 10^{-10}$	$1.16\times 10^{+02}$	$5.07\times 10^{-09}$	$3.21\times 10^{-01}$

**Table:** (g) distance—*L*

	*h*_−2_	*h*_−1_	*h*_0_	*h*_1_	*h*_2_	*q_c_*_1_	*T_c_*_1_	*q_c_*_2_	*T_c_*_2_	*q_c_*_3_	*T*_3_	*q_c_*_4_	*T_c_*_4_	*As*	*f*_0_
**All**	$1.11\times 10^{-09}$	$3.69\times 10^{-16}$	$2.13\times 10^{-06}$	$3.64\times 10^{-17}$	$1.37\times 10^{-20}$	$1.63\times 10^{-02}$	$1.63\times 10^{-01}$	$1.77\times 10^{-03}$	$4.95\times 10^{+00}$	$2.78\times 10^{-04}$	$2.18\times 10^{+02}$	$6.50\times 10^{-10}$	$7.88\times 10^{+02}$	$1.56\times 10^{-03}$	$3.14\times 10^{+00}$
**8**	$1.28\times 10^{-09}$	$2.73\times 10^{-16}$	$2.21\times 10^{-06}$	$8.55\times 10^{-09}$	$1.08\times 10^{-64}$	$1.32\times 10^{-02}$	$2.20\times 10^{-01}$	$1.84\times 10^{-03}$	$5.04\times 10^{+00}$	$1.30\times 10^{-08}$	$1.93\times 10^{+02}$	$3.28\times 10^{-04}$	$2.20\times 10^{+02}$	$1.81\times 10^{-03}$	$1.44\times 10^{+01}$
**6**	$1.48\times 10^{-09}$	$4.97\times 10^{-10}$	$2.87\times 10^{-06}$	$1.05\times 10^{-08}$	$2.48\times 10^{-114}$	$1.55\times 10^{-02}$	$2.15\times 10^{-01}$	$2.02\times 10^{-03}$	$5.35\times 10^{+00}$	$2.95\times 10^{-04}$	$2.24\times 10^{+02}$	$2.22\times 10^{-04}$	$2.27\times 10^{+02}$	$9.97\times 10^{-01}$	$2.59\times 10^{+06}$
**5**	$8.19\times 10^{-10}$	$8.50\times 10^{-17}$	$4.42\times 10^{-06}$	$2.08\times 10^{-08}$	$7.00\times 10^{-19}$	$1.88\times 10^{-02}$	$2.29\times 10^{-01}$	$2.48\times 10^{-03}$	$3.67\times 10^{+00}$	$4.52\times 10^{-04}$	$3.06\times 10^{+02}$	$9.20\times 10^{-09}$	$2.26\times 10^{+03}$	$7.95\times 10^{-01}$	$8.45\times 10^{+37}$
**4**	$1.17\times 10^{-09}$	$8.99\times 10^{-10}$	$5.59\times 10^{-06}$	$1.66\times 10^{-08}$	$5.18\times 10^{-294}$	$2.46\times 10^{-02}$	$2.12\times 10^{-01}$	$2.73\times 10^{-03}$	$3.71\times 10^{+00}$	$7.55\times 10^{-05}$	$3.32\times 10^{+02}$	$4.53\times 10^{-04}$	$3.32\times 10^{+02}$	$7.65\times 10^{-01}$	$1.91\times 10^{+04}$
**3**	$5.57\times 10^{-09}$	$1.19\times 10^{-09}$	$1.03\times 10^{-05}$	$8.12\times 10^{-10}$	$2.96\times 10^{-52}$	$2.80\times 10^{-02}$	$2.19\times 10^{-01}$	$4.20\times 10^{-03}$	$4.67\times 10^{+00}$	$2.29\times 10^{-04}$	$9.60\times 10^{+02}$	$3.80\times 10^{-04}$	$1.11\times 10^{+03}$	$7.18\times 10^{-01}$	$6.24\times 10^{+03}$
**2**	$9.01\times 10^{-09}$	$1.14\times 10^{-11}$	$1.73\times 10^{-05}$	$1.54\times 10^{-08}$	$1.21\times 10^{-36}$	$3.86\times 10^{-02}$	$2.30\times 10^{-01}$	$9.01\times 10^{-03}$	$4.29\times 10^{+00}$	$2.99\times 10^{-06}$	$5.32\times 10^{+02}$	$8.25\times 10^{-04}$	$1.94\times 10^{+03}$	$5.27\times 10^{-02}$	$6.47\times 10^{+27}$

Table A2 Estimated noise parameters of Bonita cameras type for 2..10 devices.

**Table:** (a) *x*_1_

	*h*_−2_	*h*_−1_	*h*_0_	*h*_1_	*h*_2_	*q_c_*_1_	*T_c_*_1_	*q_c_*_2_	*T_c_*_2_	*q_c_*_3_	*T*_3_
**All**	$1.51\times 10^{-07}$	$5.10\times 10^{-14}$	$1.08\times 10^{-04}$	$4.69\times 10^{-07}$	$9.44\times 10^{-24}$	$2.03\times 10^{-01}$	$4.23\times 10^{-02}$	$8.49\times 10^{-07}$	$4.32\times 10^{+02}$	$5.23\times 10^{-03}$	$5.84\times 10^{+02}$
**8**	$4.22\times 10^{-07}$	$6.06\times 10^{-14}$	$2.57\times 10^{-04}$	$4.45\times 10^{-15}$	$7.44\times 10^{-154}$	$8.85\times 10^{-02}$	$1.24\times 10^{-01}$	$1.43\times 10^{-06}$	$2.88\times 10^{+02}$	$6.70\times 10^{-03}$	$3.63\times 10^{+02}$
**6**	$6.41\times 10^{-07}$	$3.29\times 10^{-13}$	$2.79\times 10^{-04}$	$1.87\times 10^{-14}$	$1.63\times 10^{-16}$	$7.32\times 10^{-02}$	$1.78\times 10^{-01}$	$3.56\times 10^{-04}$	$3.66\times 10^{+02}$	$8.14\times 10^{-03}$	$3.66\times 10^{+02}$
**5**	$1.73\times 10^{-06}$	$2.04\times 10^{-06}$	$4.87\times 10^{-04}$	$1.92\times 10^{-14}$	$1.63\times 10^{-74}$	$4.14\times 10^{-02}$	$1.11\times 10^{+00}$	$1.25\times 10^{-02}$	$3.32\times 10^{+02}$	$3.48\times 10^{-06}$	$4.75\times 10^{+02}$
**4**	$2.15\times 10^{-06}$	$1.44\times 10^{-14}$	$7.99\times 10^{-04}$	$5.05\times 10^{-14}$	$1.11\times 10^{-16}$	$6.25\times 10^{-02}$	$4.36\times 10^{-01}$	$1.40\times 10^{-05}$	$3.46\times 10^{+02}$	$1.35\times 10^{-02}$	$3.41\times 10^{+02}$
**3**	$8.61\times 10^{-06}$	$1.74\times 10^{-04}$	$3.22\times 10^{-03}$	$4.35\times 10^{-15}$	$1.68\times 10^{-70}$	$3.23\times 10^{-01}$	$1.00\times 10^{-01}$	$6.61\times 10^{-06}$	$2.56\times 10^{+02}$	$3.23\times 10^{-02}$	$2.84\times 10^{+02}$
**2**	$4.81\times 10^{-06}$	$8.80\times 10^{-05}$	$2.09\times 10^{-03}$	$8.38\times 10^{-19}$	$4.38\times 10^{-52}$	$2.74\times 10^{-01}$	$1.00\times 10^{-01}$	$3.18\times 10^{-05}$	$2.90\times 10^{+02}$	$2.59\times 10^{-02}$	$2.81\times 10^{+02}$

**Table:** (b) *y*_1_

	*h*_−2_	*h*_−1_	*h*_0_	*h*_1_	*h*_2_	*q_c_*_1_	*T_c_*_1_	*q_c_*_2_	*T_c_*_2_	*q_c_*_3_	*T*_3_
**All**	$3.05\times 10^{-07}$	$3.40\times 10^{-15}$	$5.03\times 10^{-05}$	$5.84\times 10^{-08}$	$1.16\times 10^{-34}$	$6.69\times 10^{-02}$	$4.23\times 10^{-02}$	$8.00\times 10^{-03}$	$1.00\times 10^{+00}$	$1.89\times 10^{-03}$	$2.80\times 10^{+02}$
**8**	$5.37\times 10^{-07}$	$1.13\times 10^{-12}$	$5.22\times 10^{-05}$	$8.25\times 10^{-07}$	$3.69\times 10^{-15}$	$4.38\times 10^{-01}$	$1.05\times 10^{-02}$	$1.14\times 10^{-02}$	$1.27\times 10^{+00}$	$2.08\times 10^{-03}$	$2.13\times 10^{+02}$
**6**	$5.89\times 10^{-07}$	$1.64\times 10^{-14}$	$7.07\times 10^{-05}$	$3.66\times 10^{-07}$	$6.40\times 10^{-15}$	$2.39\times 10^{-01}$	$1.53\times 10^{-02}$	$1.43\times 10^{-02}$	$1.43\times 10^{+00}$	$3.84\times 10^{-03}$	$2.13\times 10^{+02}$
**5**	$3.05\times 10^{-07}$	$1.87\times 10^{-14}$	1.10$1.10\times 10^{-04}$	$1.24\times 10^{-13}$	$3.13\times 10^{-73}$	$7.00\times 10^{-02}$	$1.00\times 10^{-02}$	$2.04\times 10^{-02}$	$1.96\times 10^{+00}$	$7.53\times 10^{-03}$	$2.39\times 10^{+02}$
**4**	$1.52\times 10^{-06}$	$2.15\times 10^{-14}$	$1.15\times 10^{-04}$	$1.73\times 10^{-06}$	$4.07\times 10^{-16}$	$7.00\times 10^{-01}$	$1.00\times 10^{-02}$	$2.75\times 10^{-02}$	$1.75\times 10^{+00}$	$8.78\times 10^{-03}$	$2.19\times 10^{+02}$
**3**	$1.67\times 10^{-06}$	$4.26\times 10^{-12}$	$6.83\times 10^{-04}$	$12.03\times 10^{-12}$	$1.82\times 10^{-15}$	$1.00\times 10^{+00}$	$1.44\times 10^{-02}$	$3.97\times 10^{-02}$	$1.26\times 10^{+00}$	$2.01\times 10^{-02}$	$3.82\times 10^{+02}$
**2**	$3.83\times 10^{-06}$	$1.16\times 10^{-12}$	$1.04\times 10^{-03}$	$2.15\times 10^{-13}$	$1.69\times 10^{-31}$	$1.00\times 10^{+00}$	$1.89\times 10^{-02}$	$3084\times 10^{-02}$	$1.30\times 10^{+00}$	$2.08\times 10^{-02}$	$3.72\times 10^{+02}$

**Table:** (c) *z*_1_

	*h*_−2_	*h*_−1_	*h*_0_	*h*_1_	*h*_2_	*q_c_*_1_	*T_c_*_1_	*q_c_*_2_	*T_c_*_2_	*q_c_*_3_	*T*_3_	*q_c_*_4_	*T_c_*_4_
**All**	$1.58\times 10^{-07}$	$1.14\times 10^{-14}$	$8.87\times 10^{-05}$	$2.07\times 10^{-14}$	$2.67\times 10^{-231}$	$1.12\times 10^{-01}$	$6.35\times 10^{-02}$	$7.39\times 10^{-08}$	$2.01\times 10^{+00}$	$2.59\times 10^{-05}$	$4.44\times 10^{+02}$	$4.81\times 10^{-03}$	$4.44\times 10^{+02}$
**8**	$2.24\times 10^{-07}$	$5.38\times 10^{-13}$	$1.37\times 10^{-04}$	$3.07\times 10^{-07}$	$4.51\times 10^{-135}$	$1.82\times 10^{-01}$	$3.85\times 10^{-02}$	$1.50\times 10^{-02}$	$1.00\times 10^{+00}$	$5.96\times 10^{-03}$	$3.46\times 10^{+02}$	$2.46\times 10^{-07}$	$1.39\times 10^{+03}$
**6**	$1.79\times 10^{-07}$	$5.87\times 10^{-14}$	$7.45\times 10^{-05}$	$1.30\times 10^{-13}$	$1.02\times 10^{-53}$	$9.75\times 10^{-02}$	$7.88\times 10^{-02}$	$1.40\times 10^{-07}$	$4.62\times 10^{+00}$	$1.01\times 10^{-03}$	$5.00\times 10^{+02}$	$4.86\times 10^{-03}$	$5.00\times 10^{+02}$
**5**	$2.14\times 10^{-07}$	$1.81\times 10^{-14}$	$8.08\times 10^{-05}$	$4.81\times 10^{-14}$	$1.17\times 10^{-16}$	$1.13\times 10^{-01}$	$6.13\times 10^{-02}$	$3.04\times 10^{-03}$	$1.00\times 10^{+00}$	$2.75\times 10^{-07}$	$2.72\times 10^{+02}$	$5.66\times 10^{-03}$	$3.04\times 10^{+02}$
**4**	$8.68\times 10^{-07}$	$1.47\times 10^{-14}$	$1.47\times 10^{-04}$	$1.39\times 10^{-13}$	$1.26\times 10^{-75}$	$1.68\times 10^{-01}$	$4.97\times 10^{-02}$	$1.40\times 10^{-02}$	$1.00\times 10^{+00}$	$6.90\times 10^{-06}$	$3.58\times 10^{+02}$	$8.86\times 10^{-03}$	$3.41\times 10^{+02}$
**3**	$1.11\times 10^{-06}$	$5.33\times 10^{-19}$	$4.60\times 10^{-04}$	$7.17\times 10^{-18}$	$4.83\times 10^{-55}$	$1.00\times 10^{+00}$	$1.74\times 10^{-02}$	$4.29\times 10^{-07}$	$3.33\times 10^{+00}$	$3.79\times 10^{-06}$	$3.46\times 10^{+02}$	$1.15\times 10^{-02}$	$3.42\times 10^{+02}$
**2**	$1.63\times 10^{-06}$	$2.21\times 10^{-13}$	$8.59\times 10^{-04}$	$1.76\times 10^{-13}$	$1.46\times 10^{-124}$	$1.00\times 10^{+00}$	$1.91\times 10^{-02}$	$2.25\times 10^{-07}$	$3.42\times 10^{+00}$	$1.18\times 10^{-06}$	$9.87\times 10^{+02}$	$1.49\times 10^{-02}$	$3.08\times 10^{+02}$

**Table:** (d) *x*_2_

	*h*_−2_	*h*_−1_	*h*_0_	*h*_1_	*h*_2_	*q_c_*_1_	*T_c_*_1_	*q_c_*_2_	*T_c_*_2_	*q_c_*_3_	*T*_3_
**All**	$2.84\times 10^{-08}$	$1.72\times 10^{-15}$	$3.75\times 10^{-05}$	$4.99\times 10^{-16}$	$6.55\times 10^{-278}$	$6.39\times 10^{-02}$	$9.30\times 10^{-02}$	$2.06\times 10^{-07}$	$1.96\times 10^{+02}$	$3.80\times 10^{-03}$	$2.89\times 10^{+02}$
**8**	$2.60\times 10^{-08}$	$2.22\times 10^{-21}$	$6.59\times 10^{-05}$	$2.49\times 10^{-17}$	$5.03\times 10^{-136}$	$8.46\times 10^{-02}$	$9.43\times 10^{-02}$	$5.75\times 10^{-07}$	$1.93\times 10^{+02}$	$5.32\times 10^{-03}$	$2.34\times 10^{+02}$
**6**	$1.26\times 10^{-07}$	$2.89\times 10^{-06}$	$9.24\times 10^{-05}$	$6.32\times 10^{-17}$	$7.76\times 10^{-23}$	$1.01\times 10^{-01}$	$1.18\times 10^{-01}$	$5.15\times 10^{-06}$	$1.86\times 10^{+02}$	$7.25\times 10^{-03}$	$1.82\times 10^{+02}$
**5**	$2.56\times 10^{-07}$	$5.80\times 10^{-16}$	$8.06\times 10^{-05}$	$3.26\times 10^{-13}$	$2.45\times 10^{-10}$	$2.02\times 10^{-01}$	$4.48\times 10^{-02}$	$4.89\times 10^{-08}$	$1.96\times 10^{+02}$	$3.50\times 10^{-03}$	$2.22\times 10^{+02}$
**4**	$2.28\times 10^{-07}$	$1.87\times 10^{-13}$	$1.97\times 10^{-04}$	$1.48\times 10^{-15}$	$1.24\times 10^{-29}$	$1.48\times 10^{-01}$	$1.07\times 10^{-01}$	$2.77\times 10^{-07}$	$1.78\times 10^{+02}$	$9.28\times 10^{-03}$	$1.85\times 10^{+02}$
**3**	$2.84\times 10^{-07}$	$3.01\times 10^{-04}$	$7.19\times 10^{-04}$	$1.58\times 10^{-12}$	$2.72\times 10^{-20}$	$4.52\times 10^{-01}$	$5.79\times 10^{-02}$	$3.65\times 10^{-02}$	$5.95\times 10^{+01}$	$7.31\times 10^{-03}$	$9.28\times 10^{+02}$
**2**	$1.23\times 10^{-06}$	$7.34\times 10^{-04}$	$7.60\times 10^{-04}$	$3.99\times 10^{-06}$	$9.54\times 10^{-48}$	$7.65\times 10^{-01}$	$2.94\times 10^{-02}$	$6.96\times 10^{-02}$	$3.21\times 10^{+01}$	$1.87\times 10^{-02}$	$2.63\times 10^{+02}$

**Table:** (e) *y*_2_

	*h*_−2_	*h*_−1_	*h*_0_	*h*_1_	*h*_2_	*q_c_*_1_	*T_c_*_1_	*q_c_*_2_	*T_c_*_2_	*q_c_*_3_	*T*_3_
**All**	$2.49\times 10^{-07}$	$8.04\times 10^{-16}$	$2.97\times 10^{-05}$	$1.15\times 10^{-16}$	$9.63\times 10^{-203}$	$6.98\times 10^{-02}$	$9.21\times 10^{-02}$	$4.87\times 10^{-03}$	$5.87\times 10^{+01}$	$6.49\times 10^{-10}$	$7.55\times 10^{+02}$
**8**	$3.58\times 10^{-07}$	$1.49\times 10^{-15}$	$4.00\times 10^{-05}$	$5.21\times 10^{-17}$	$1.23\times 10^{-19}$	$6.87\times 10^{-02}$	$9.73\times 10^{-02}$	$3.83\times 10^{-03}$	$5.96\times 10^{+01}$	$1.26\times 10^{-09}$	$5.54\times 10^{+02}$
**6**	$3.55\times 10^{-07}$	$3.23\times 10^{-16}$	$6.27\times 10^{-05}$	$8.38\times 10^{-17}$	$1.34\times 10^{-84}$	$1.01\times 10^{-01}$	$9.35\times 10^{-02}$	$7.53\times 10^{-03}$	$5.43\times 10^{+01}$	$1.51\times 10^{-08}$	$6.63\times 10^{+02}$
**5**	$6.67\times 10^{-08}$	$7.32\times 10^{-35}$	$4.09\times 10^{-05}$	$4.11\times 10^{-14}$	$8.14\times 10^{-56}$	$9.46\times 10^{-02}$	$5.76\times 10^{-02}$	$2.30\times 10^{-07}$	$2.40\times 10^{+02}$	$2.46\times 10^{-03}$	$8.78\times 10^{+02}$
**4**	$4.91\times 10^{-07}$	$1.26\times 10^{-15}$	$9.85\times 10^{-05}$	$4.51\times 10^{-17}$	$1.74\times 10^{-80}$	$1.39\times 10^{-01}$	$8.15\times 10^{-02}$	$7.58\times 10^{-03}$	$6.38\times 10^{+01}$	$4.36\times 10^{-09}$	$4.58\times 10^{+02}$
**3**	$1.17\times 10^{-07}$	$2.90\times 10^{-13}$	$2.36\times 10^{-04}$	$8.80\times 10^{-14}$	$2.58\times 10^{-64}$	$2.10\times 10^{-01}$	$8.00\times 10^{-02}$	$1.28\times 10^{-02}$	$6.35\times 10^{+01}$	$2.52\times 10^{-03}$	$1.00\times 10^{+03}$
**2**	$2.75\times 10^{-07}$	$5.17\times 10^{-05}$	$3.92\times 10^{-04}$	$4.11\times 10^{-13}$	$1.22\times 10^{-16}$	$2.50\times 10^{-01}$	$7.43\times 10^{-02}$	$2.18\times 10^{-02}$	$5.30\times 10^{+01}$	$3.78\times 10^{-03}$	$6.58\times 10^{+02}$

**Table:** (f) *z*_2_

	*h*_−2_	*h*_−1_	*h*_0_	*h*_1_	*h*_2_	*q_c_*_1_	*T_c_*_1_	*q_c_*_2_	*T_c_*_2_	*q_c_*_3_	*T*_3_	*q_c_*_4_	*T*_4_
**All**	$1.09\times 10^{-07}$	$2.60\times 10^{-16}$	$2.66\times 10^{-05}$	$5.01\times 10^{-15}$	$1.27\times 10^{-17}$	$6.71\times 10^{-02}$	$7.44\times 10^{-02}$	$1.92\times 10^{-08}$	$3.79\times 10^{+00}$	$2.06\times 10^{-07}$	$3.26\times 10^{+02}$	$4.26\times 10^{-03}$	$2.72\times 10^{+02}$
**8**	$7.80\times 10^{-08}$	$4.66\times 10^{-16}$	$4.29\times 10^{-05}$	$2.39\times 10^{-14}$	$6.73\times 10^{-17}$	$8.26\times 10^{-02}$	$6.47\times 10^{-02}$	$9.21\times 10^{-08}$	$1.90\times 10^{+00}$	$5.16\times 10^{-07}$	$2.99\times 10^{+02}$	$4.56\times 10^{-03}$	$3.49\times 10^{+02}$
**6**	$9.31\times 10^{-08}$	$8.69\times 10^{-32}$	$3.32\times 10^{-05}$	$8.88\times 10^{-19}$	$1.54\times 10^{-41}$	$5.64\times 10^{-02}$	$8.44\times 10^{-02}$	$1.41\times 10^{-07}$	$2.41\times 10^{+00}$	$3.86\times 10^{-07}$	$3.71\times 10^{+02}$	$3.98\times 10^{-03}$	$3.56\times 10^{+02}$
**5**	$1.25\times 10^{-17}$	$5.20\times 10^{-14}$	$2.47\times 10^{-05}$	$3.35\times 10^{-07}$	$1.92\times 10^{-15}$	$1.90\times 10^{-01}$	$2.02\times 10^{-02}$	$2.22\times 10^{-07}$	$2.62\times 10^{+00}$	$6.31\times 10^{-07}$	$9.16\times 10^{+02}$	$2.85\times 10^{-03}$	$1.39\times 10^{+03}$
**4**	$2.27\times 10^{-07}$	$1.22\times 10^{-27}$	$6.65\times 10^{-05}$	$7.89\times 10^{-08}$	$2.24\times 10^{-16}$	$8.96\times 10^{-02}$	$6.13\times 10^{-02}$	$1.72\times 10^{-08}$	$6.01\times 10^{+00}$	$4.48\times 10^{-03}$	$2.41\times 10^{+02}$	$8.49\times 10^{-08}$	$1.32\times 10^{+03}$
**3**	$3.29\times 10^{-07}$	$2.29\times 10^{-14}$	$2.18\times 10^{-04}$	$1.37\times 10^{-14}$	$1.90\times 10^{-17}$	$1.06\times 10^{-01}$	$1.26\times 10^{-01}$	$1.84\times 10^{-07}$	$2.95\times 10^{+00}$	$1.04\times 10^{-07}$	$3.77\times 10^{+01}$	$9.09\times 10^{-03}$	$1.83\times 10^{+02}$
**2**	$5.91\times 10^{-07}$	$6.08\times 10^{-12}$	$4.52\times 10^{-04}$	$5.51\times 10^{-13}$	$1.14\times 10^{-80}$	$1.93\times 10^{-01}$	$9.98\times 10^{-02}$	$1.97\times 10^{-02}$	$1.00\times 10^{+00}$	$6.15\times 10^{-03}$	$2.67\times 10^{+02}$	$1.72\times 10^{-02}$	$1.00\times 10^{+02}$

**Table:** (g) distance—*L*

	*h*_−2_	*h*_−1_	*h*_0_	*h*_1_	*h*_2_	*q_c_*_1_	*T_c_*_1_	*q_c_*_2_	*T_c_*_2_	*q_c_*_3_	*T*_3_
**All**	$3.66\times 10^{-07}$	$1.07\times 10^{-14}$	$1.42\times 10^{-04}$	$9.68\times 10^{-07}$	$1.64\times 10^{-57}$	$2.71\times 10^{-01}$	$3.80\times 10^{-02}$	$3.60\times 10^{-03}$	$5.77\times 10^{+02}$	$4.54\times 10^{-03}$	$6.29\times 10^{+02}$
**8**	$4.76\times 10^{-07}$	$1.65\times 10^{-14}$	$3.66\times 10^{-04}$	$6.23\times 10^{-07}$	$7.35\times 10^{-134}$	$2.54\times 10^{-01}$	$4.96\times 10^{-02}$	$6.34\times 10^{-08}$	$7.52\times 10^{+02}$	$5.71\times 10^{-03}$	$5.56\times 10^{+02}$
**6**	$8.36\times 10^{-07}$	$2.17\times 10^{-14}$	$4.73\times 10^{-04}$	$4.08\times 10^{-14}$	$1.44\times 10^{-281}$	$2.32\times 10^{-01}$	$7.01\times 10^{-02}$	$4.49\times 10^{-07}$	$6.75\times 10^{+02}$	$7.86\times 10^{-03}$	$3.67\times 10^{+02}$
**5**	$1.67\times 10^{-06}$	$6.51\times 10^{-13}$	$1.09\times 10^{-03}$	$9.42\times 10^{-14}$	$8.56\times 10^{-85}$	$1.23\times 10^{-01}$	$1.65\times 10^{-01}$	$1.35\times 10^{-06}$	$4.90\times 10^{+02}$	$1.09\times 10^{-02}$	$3.77\times 10^{+02}$
**4**	$2.13\times 10^{-06}$	$7.89\times 10^{-14}$	$1.29\times 10^{-03}$	$4.91\times 10^{-13}$	$7.31\times 10^{-101}$	$3.31\times 10^{-01}$	$7.02\times 10^{-02}$	$9.45\times 10^{-06}$	$5.40\times 10^{+02}$	$1.21\times 10^{-02}$	$5.22\times 10^{+02}$
**3**	$1.78\times 10^{-05}$	$2.76\times 10^{-04}$	$4.52\times 10^{-03}$	$2.25\times 10^{-14}$	$5.49\times 10^{-79}$	$1.00\times 10^{+00}$	$4.68\times 10^{-02}$	$3.76\times 10^{-02}$	$8.31\times 10^{+01}$	$2.27\times 10^{-02}$	$2.00\times 10^{+02}$
**2**	$1.10\times 10^{-05}$	$4.28\times 10^{-04}$	$4.09\times 10^{-03}$	$1.84\times 10^{-13}$	$1.29\times 10^{-38}$	$1.00\times 10^{+00}$	$4.81\times 10^{-02}$	$6.18\times 10^{-02}$	$2.92\times 10^{+01}$	$3.17\times 10^{-02}$	$2.00\times 10^{+02}$

Table A3 Estimated noise parameters of Vantage cameras type for 2..9 devices.

**Table:** (a) *x*_1_

	*h*_−2_	*h*_−1_	*h*_0_	*h*_1_	*h*_2_	*q_c_*_1_	*T_c_*_1_	*q_c_*_2_	*T_c_*_2_	*q_c_*_3_	*T*_3_
**All**	$6.91\times 10^{-08}$	$7.58\times 10^{-14}$	$3.21\times 10^{-05}$	$2.33\times 10^{-12}$	$2.26\times 10^{-10}$	$6.88\times 10^{-02}$	$6.10\times 10^{-02}$	$5.97\times 10^{-08}$	$5.30\times 10^{+01}$	$6.38\times 10^{-04}$	$2.00\times 10^{+03}$
**9**	$1.68\times 10^{-08}$	$9.54\times 10^{-07}$	$2.64\times 10^{-05}$	$1.05\times 10^{-10}$	$1.98\times 10^{-10}$	$4.00\times 10^{-02}$	$5.94\times 10^{-02}$	$1.05\times 10^{-03}$	$1.47\times 10^{+01}$	$8.34\times 10^{-04}$	$2.51\times 10^{+02}$
**8**	$1.33\times 10^{-17}$	$1.40\times 10^{-13}$	$1.39\times 10^{-05}$	$8.82\times 10^{-13}$	$6.45\times 10^{-11}$	$1.92\times 10^{-02}$	$9.99\times 10^{-02}$	$1.51\times 10^{-03}$	$9.34\times 10^{+00}$	$1.36\times 10^{-03}$	$8.02\times 10^{+02}$
**6**	$2.38\times 10^{-08}$	$2.39\times 10^{-11}$	$3.70\times 10^{-05}$	$2.41\times 10^{-11}$	$2.38\times 10^{-10}$	$3.78\times 10^{-02}$	$6.77\times 10^{-02}$	$1.51\times 10^{-03}$	$6.57\times 10^{+00}$	$8.53\times 10^{-04}$	$2.71\times 10^{+02}$
**5**	$4.34\times 10^{-08}$	$8.09\times 10^{-07}$	$4.65\times 10^{-05}$	$1.66\times 10^{-10}$	$3.94\times 10^{-10}$	$5.37\times 10^{-02}$	$5.18\times 10^{-02}$	$1.28\times 10^{-03}$	$8.20\times 10^{+00}$	$6.46\times 10^{-04}$	$1.10\times 10^{+03}$
**4**	$1.11\times 10^{-08}$	$3.70\times 10^{-06}$	$2.51\times 10^{-05}$	$1.64\times 10^{-07}$	$2.28\times 10^{-189}$	$3.79\times 10^{-01}$	$1.59\times 10^{-02}$	$1.81\times 10^{-03}$	$7.49\times 10^{+00}$	$1.35\times 10^{-03}$	$8.45\times 10^{+02}$
**3**	$1.66\times 10^{-06}$	$6.36\times 10^{-20}$	$1.17\times 10^{-04}$	$3.86\times 10^{-07}$	$1.13\times 10^{-21}$	$1.39\times 10^{-02}$	$1.13\times 10^{-01}$	$7.65\times 10^{-11}$	$1.84\times 10^{+01}$	$4.92\times 10^{-03}$	$4.07\times 10^{+00}$
**2**	$1.22\times 10^{-06}$	$3.07\times 10^{-20}$	$4.51\times 10^{-04}$	$3.50\times 10^{-06}$	$1.77\times 10^{-77}$	$7.21\times 10^{-08}$	$1.15\times 10^{-02}$	$2.23\times 10^{-02}$	$1.80\times 10^{+00}$	$2.28\times 10^{-11}$	$1.09\times 10^{+03}$

**Table:** (b) *y*_1_

	*h*_−2_	*h*_−1_	*h*_0_	*h*_1_	*h*_2_	*q_c_*_1_	*T_c_*_1_	*q_c_*_2_	*T_c_*_2_	*q_c_*_3_	*T*_3_
**All**	$3.30\times 10^{-07}$	$6.68\times 10^{-13}$	$4.46\times 10^{-05}$	$1.52\times 10^{-06}$	$2.29\times 10^{-14}$	$1.89\times 10^{-01}$	$2.50\times 10^{-02}$	$1.38\times 10^{-07}$	$8.61\times 10^{+01}$	$6.44\times 10^{-08}$	$1.78\times 10^{+03}$
**9**	$3.04\times 10^{-07}$	$1.53\times 10^{-21}$	$3.04\times 10^{-05}$	$1.74\times 10^{-20}$	$3.43\times 10^{-10}$	$3.21\times 10^{-02}$	$8.13\times 10^{-02}$	$1.69\times 10^{-11}$	$7.71\times 10^{+01}$	$1.74\times 10^{-11}$	$1.81\times 10^{+03}$
**8**	$3.43\times 10^{-07}$	$1.22\times 10^{-19}$	$1.17\times 10^{-05}$	$1.29\times 10^{-07}$	$7.44\times 10^{-11}$	$5.81\times 10^{-03}$	$6.82\times 10^{-01}$	$6.01\times 10^{-11}$	$6.48\times 10^{+01}$	$4.49\times 10^{-12}$	$1.45\times 10^{+03}$
**6**	$3.87\times 10^{-07}$	$1.13\times 10^{-19}$	$3.63\times 10^{-05}$	$9.38\times 10^{-07}$	$6.77\times 10^{-18}$	$1.74\times 10^{-01}$	$1.91\times 10^{-02}$	$4.16\times 10^{-09}$	$3.93\times 10^{+01}$	$7.51\times 10^{-08}$	$7.81\times 10^{+02}$
**5**	$3.53\times 10^{-07}$	$1.38\times 10^{-21}$	$2.76\times 10^{-05}$	$9.58\times 10^{-07}$	$2.70\times 10^{-126}$	$2.18\times 10^{-01}$	$1.69\times 10^{-02}$	$1.72\times 10^{-11}$	$5.26\times 10^{+01}$	$1.69\times 10^{-11}$	$1.46\times 10^{+03}$
**4**	$5.25\times 10^{-07}$	$1.10\times 10^{-19}$	$1.30\times 10^{-05}$	$5.23\times 10^{-07}$	$5.64\times 10^{-21}$	$3.29\times 10^{-01}$	$1.00\times 10^{-02}$	$1.69\times 10^{-10}$	$6.65\times 10^{+01}$	$5.12\times 10^{-11}$	$1.70\times 10^{+03}$
**3**	$1.69\times 10^{-14}$	$1.79\times 10^{-14}$	$1.84\times 10^{-04}$	$5.32\times 10^{-07}$	$3.87\times 10^{-108}$	$1.38\times 10^{-04}$	$1.39\times 10^{-02}$	$4.97\times 10^{-03}$	$7.88\times 10^{+00}$	$6.51\times 10^{-03}$	$1.66\times 10^{+03}$
**2**	$1.55\times 10^{-06}$	$5.81\times 10^{-13}$	$5.79\times 10^{-03}$	$4.74\times 10^{-11}$	$2.05\times 10^{-08}$	$2.86\times 10^{-02}$	$1.00\times 10^{+00}$	$1.16\times 10^{-02}$	$5.00\times 10^{+00}$	$6.45\times 10^{-03}$	$2.00\times 10^{+03}$

**Table:** (c) *z*_1_

	*h*_−2_	*h*_−1_	*h*_0_	*h*_1_	*h*_2_	*q_c_*_1_	*T_c_*_1_	*q_c_*_2_	*T_c_*_2_	*q_c_*_3_	*T*_3_
**All**	$5.06\times 10^{-08}$	$7.72\times 10^{-06}$	$1.96\times 10^{-04}$	$1.28\times 10^{-06}$	$1.12\times 10^{-09}$	$1.83\times 10^{-01}$	$3.52\times 10^{-02}$	$3.07\times 10^{-03}$	$1.38\times 10^{+01}$	$1.69\times 10^{-03}$	$3.83\times 10^{+02}$
**9**	$5.72\times 10^{-08}$	$7.29\times 10^{-14}$	$1.01\times 10^{-04}$	$1.95\times 10^{-15}$	$1.19\times 10^{-41}$	$8.53\times 10^{-03}$	$2.41\times 10^{+00}$	$2.84\times 10^{-03}$	$1.39\times 10^{+01}$	$1.95\times 10^{-03}$	$2.42\times 10^{+02}$
**8**	$7.38\times 10^{-27}$	$5.85\times 10^{-13}$	$2.86\times 10^{-05}$	$4.07\times 10^{-17}$	$3.49\times 10^{-55}$	$1.88\times 10^{-02}$	$2.14\times 10^{-01}$	$2.67\times 10^{-03}$	$1.00\times 10^{+01}$	$2.33\times 10^{-03}$	$9.33\times 10^{+02}$
**6**	$1.40\times 10^{-07}$	$4.95\times 10^{-14}$	$2.30\times 10^{-04}$	$7.33\times 10^{-07}$	$4.49\times 10^{-17}$	$1.65\times 10^{-02}$	$1.00\times 10^{+00}$	$7.43\times 10^{-03}$	$1.00\times 10^{+01}$	$2.47\times 10^{-03}$	$4.33\times 10^{+02}$
**5**	$1.06\times 10^{-07}$	$2.03\times 10^{-15}$	$2.17\times 10^{-04}$	$3.22\times 10^{-13}$	$2.91\times 10^{-10}$	$1.21\times 10^{-02}$	$3.05\times 10^{+00}$	$4.15\times 10^{-03}$	$1.00\times 10^{+01}$	$2.88\times 10^{-03}$	$1.73\times 10^{+02}$
**4**	$3.31\times 10^{-07}$	$6.65\times 10^{-20}$	$7.48\times 10^{-05}$	$8.79\times 10^{-08}$	$6.94\times 10^{-119}$	$3.08\times 10^{-07}$	$1.63\times 10^{-02}$	$4.30\times 10^{-03}$	$1.83\times 10^{+01}$	$5.64\times 10^{-10}$	$1.78\times 10^{+03}$
**3**	$2.02\times 10^{-06}$	$5.51\times 10^{-18}$	$4.43\times 10^{-04}$	$2.32\times 10^{-06}$	$4.04\times 10^{-21}$	$2.87\times 10^{-02}$	$1.66\times 10^{-01}$	$9.05\times 10^{-03}$	$1.58\times 10^{+01}$	$2.75\times 10^{-10}$	$1.18\times 10^{+03}$
**2**	$9.78\times 10^{-07}$	$4.27\times 10^{-13}$	$2.75\times 10^{-03}$	$2.01\times 10^{-06}$	$1.85\times 10^{-08}$	$1.44\times 10^{-02}$	$7.88\times 10^{-01}$	$1.09\times 10^{-02}$	$1.00\times 10^{+01}$	$3.09\times 10^{-03}$	$2.00\times 10^{+03}$

**Table:** (d) *x*_2_

	*h*_−2_	*h*_−1_	*h*_0_	*h*_1_	*h*_2_	*q_c_*_1_	*T_c_*_1_	*q_c_*_2_	*T_c_*_2_	*q_c_*_3_	*T*_3_
**All**	$3.57\times 10^{-07}$	$1.72\times 10^{-18}$	$2.09\times 10^{-05}$	$8.51\times 10^{-07}$	$2.86\times 10^{-20}$	$2.88\times 10^{-01}$	$4.36\times 10^{-02}$	$4.83\times 10^{-03}$	$5.00\times 10^{+00}$	$2.06\times 10^{-10}$	$7.50\times 10^{+02}$
**9**	$7.36\times 10^{-08}$	$2.58\times 10^{-06}$	$1.38\times 10^{-05}$	$5.75\times 10^{-08}$	$5.97\times 10^{-11}$	$6.82\times 10^{-02}$	$2.91\times 10^{-02}$	$1.36\times 10^{-03}$	$6.69\times 10^{+01}$	$1.45\times 10^{-03}$	$1.00\times 10^{+01}$
**8**	$7.30\times 10^{-08}$	$7.31\times 10^{-16}$	$2.34\times 10^{-05}$	$7.52\times 10^{-08}$	$8.97\times 10^{-18}$	$5.39\times 10^{-03}$	$5.85\times 10^{-01}$	$2.28\times 10^{-03}$	$1.70\times 10^{+01}$	$6.37\times 10^{-04}$	$2.27\times 10^{+02}$
**6**	$5.07\times 10^{-08}$	$3.29\times 10^{-06}$	$2.33\times 10^{-05}$	$2.94\times 10^{-07}$	$6.57\times 10^{-40}$	$1.34\times 10^{-01}$	$2.01\times 10^{-02}$	$1.87\times 10^{-03}$	$1.09\times 10^{+01}$	$1.39\times 10^{-03}$	$1.27\times 10^{+02}$
**5**	$1.78\times 10^{-08}$	$3.14\times 10^{-06}$	$1.58\times 10^{-05}$	$3.45\times 10^{-07}$	$1.01\times 10^{-14}$	$1.48\times 10^{-01}$	$2.34\times 10^{-02}$	$2.97\times 10^{-07}$	$7.45\times 10^{+01}$	$2.26\times 10^{-03}$	$1.20\times 10^{+02}$
**4**	$7.76\times 10^{-08}$	$1.07\times 10^{-21}$	$3.25\times 10^{-05}$	$1.32\times 10^{-07}$	$2.32\times 10^{-45}$	$2.75\times 10^{-03}$	$7.26\times 10^{-01}$	$1.56\times 10^{-03}$	$5.00\times 10^{+00}$	$6.04\times 10^{-12}$	$3.52\times 10^{+02}$
**3**	$1.11\times 10^{-06}$	$1.02\times 10^{-19}$	$1.05\times 10^{-04}$	$8.44\times 10^{-07}$	$1.49\times 10^{-70}$	$3.31\times 10^{-02}$	$6.01\times 10^{-02}$	$4.51\times 10^{-03}$	$1.12\times 10^{+01}$	$4.19\times 10^{-10}$	$6.47\times 10^{+02}$
**2**	$1.01\times 10^{-06}$	$1.91\times 10^{-11}$	$1.06\times 10^{-04}$	$9.24\times 10^{-07}$	$1.54\times 10^{-13}$	$4.17\times 10^{-02}$	$4.87\times 10^{-02}$	$4.55\times 10^{-03}$	$1.23\times 10^{+01}$	$1.60\times 10^{-03}$	$1.00\times 10^{+03}$

**Table:** (e) *y*_2_

	*h*_−2_	*h*_−1_	*h*_0_	*h*_1_	*h*_2_	*q_c_*_1_	*T_c_*_1_	*q_c_*_2_	*T_c_*_2_	*q_c_*_3_	*T*_3_
**All**	$7.00\times 10^{-07}$	$7.81\times 10^{-20}$	$3.31\times 10^{-05}$	$1.10\times 10^{-06}$	$8.52\times 10^{-24}$	$3.27\times 10^{-01}$	$4.45\times 10^{-02}$	$5.71\times 10^{-03}$	$1.00\times 10^{+00}$	$4.57\times 10^{-11}$	$7.57\times 10^{+02}$
**9**	$2.87\times 10^{-07}$	$5.04\times 10^{-22}$	$1.73\times 10^{-05}$	$1.23\times 10^{-07}$	$1.67\times 10^{-39}$	$1.48\times 10^{-02}$	$1.04\times 10^{-01}$	$1.10\times 10^{-11}$	$5.71\times 10^{+01}$	$8.10\times 10^{-12}$	$5.13\times 10^{+02}$
**8**	$3.00\times 10^{-07}$	$1.20\times 10^{-21}$	$1.87\times 10^{-05}$	$1.80\times 10^{-07}$	$4.97\times 10^{-52}$	$9.22\times 10^{-03}$	$1.78\times 10^{-01}$	$9.94\times 10^{-12}$	$6.08\times 10^{+01}$	$1.08\times 10^{-12}$	$1.16\times 10^{+03}$
**6**	$5.70\times 10^{-07}$	$1.77\times 10^{-21}$	$5.10\times 10^{-05}$	$3.32\times 10^{-07}$	$4.11\times 10^{-23}$	$1.63\times 10^{-02}$	$1.24\times 10^{-01}$	$1.84\times 10^{-11}$	$3.15\times 10^{+01}$	$1.84\times 10^{-11}$	$4.73\times 10^{+02}$
**5**	$6.18\times 10^{-07}$	$1.18\times 10^{-21}$	$5.69\times 10^{-05}$	$2.54\times 10^{-07}$	$1.94\times 10^{-22}$	$2.13\times 10^{-02}$	$1.18\times 10^{-01}$	$1.75\times 10^{-11}$	$3.72\times 10^{+01}$	$2.41\times 10^{-12}$	$1.52\times 10^{+03}$
**4**	$7.42\times 10^{-07}$	$3.67\times 10^{-22}$	$5.73\times 10^{-05}$	$4.83\times 10^{-07}$	$5.82\times 10^{-23}$	$6.66\times 10^{-03}$	$1.40\times 10^{-01}$	$1.49\times 10^{-11}$	$6.75\times 10^{+01}$	$2.22\times 10^{-12}$	$9.24\times 10^{+02}$
**3**	$3.43\times 10^{-14}$	$7.65\times 10^{-13}$	$1.48\times 10^{-04}$	$1.29\times 10^{-06}$	$5.13\times 10^{-42}$	$9.16\times 10^{-02}$	$2.34\times 10^{-02}$	$4.17\times 10^{-03}$	$1.22\times 10^{+01}$	$5.63\times 10^{-03}$	$1.50\times 10^{+03}$
**2**	$1.56\times 10^{-55}$	$3.38\times 10^{-12}$	$1.51\times 10^{-04}$	$1.34\times 10^{-06}$	$2.72\times 10^{-14}$	$6.62\times 10^{-02}$	$3.32\times 10^{-02}$	$4.05\times 10^{-03}$	$1.07\times 10^{+01}$	$5.59\times 10^{-03}$	$1.84\times 10^{+03}$

**Table:** (f) *z*_2_

	*h*_−2_	*h*_−1_	*h*_0_	*h*_1_	*h*_2_	*q_c_*_1_	*T_c_*_1_	*q_c_*_2_	*T_c_*_2_	*q_c_*_3_	*T*_3_
**All**	$8.45\times 10^{-07}$	$1.28\times 10^{-17}$	$4.79\times 10^{-05}$	$2.33\times 10^{-06}$	$1.28\times 10^{-20}$	$4.92\times 10^{-01}$	$4.36\times 10^{-02}$	$8.16\times 10^{-03}$	$5.60\times 10^{+00}$	$4.33\times 10^{-10}$	$1.57\times 10^{+02}$
**9**	$1.58\times 10^{-08}$	$1.06\times 10^{-13}$	$3.09\times 10^{-05}$	$9.29\times 10^{-13}$	$1.17\times 10^{-10}$	$1.18\times 10^{-02}$	$2.09\times 10^{-01}$	$2.35\times 10^{-03}$	$1.55\times 10^{+01}$	$2.00\times 10^{-03}$	$3.84\times 10^{+02}$
**8**	$6.99\times 10^{-08}$	$1.04\times 10^{-13}$	$3.99\times 10^{-05}$	$3.74\times 10^{-07}$	$1.68\times 10^{-69}$	$9.82\times 10^{-03}$	$2.64\times 10^{-01}$	$3.00\times 10^{-03}$	$2.49\times 10^{+01}$	$1.54\times 10^{-03}$	$4.91\times 10^{+02}$
**6**	$1.93\times 10^{-07}$	$4.51\times 10^{-15}$	$8.39\times 10^{-05}$	$4.72\times 10^{-07}$	$5.47\times 10^{-17}$	$2.16\times 10^{-02}$	$1.30\times 10^{-01}$	$3.59\times 10^{-03}$	$2.99\times 10^{+01}$	$2.02\times 10^{-03}$	$3.06\times 10^{+02}$
**5**	$9.92\times 10^{-08}$	$5.29\times 10^{-14}$	$7.82\times 10^{-05}$	$3.02\times 10^{-07}$	$3.46\times 10^{-16}$	$1.85\times 10^{-02}$	$1.63\times 10^{-01}$	$3.35\times 10^{-03}$	$2.30\times 10^{+01}$	$2.61\times 10^{-03}$	$3.95\times 10^{+02}$
**4**	$4.74\times 10^{-07}$	$2.78\times 10^{-14}$	$8.11\times 10^{-05}$	$7.05\times 10^{-07}$	$2.31\times 10^{-16}$	$5.26\times 10^{-03}$	$1.21\times 10^{-01}$	$3.92\times 10^{-03}$	$2.74\times 10^{+01}$	$1.04\times 10^{-03}$	$1.00\times 10^{+03}$
**3**	$6.74\times 10^{-07}$	$1.33\times 10^{-15}$	$3.74\times 10^{-04}$	$3.40\times 10^{-06}$	$1.25\times 10^{-121}$	$5.36\times 10^{-02}$	$6.81\times 10^{-02}$	$5.85\times 10^{-03}$	$1.34\times 10^{+01}$	$5.67\times 10^{-03}$	$9.60\times 10^{+01}$
**2**	$6.99\times 10^{-07}$	$2.22\times 10^{-16}$	$3.73\times 10^{-04}$	$3.39\times 10^{-06}$	$1.27\times 10^{-152}$	$5.42\times 10^{-02}$	$6.73\times 10^{-02}$	$5.85\times 10^{-03}$	$1.39\times 10^{+01}$	$5.69\times 10^{-03}$	$9.69\times 10^{+01}$

**Table:** (g) distance—*L*

	*h*_−2_	*h*_−1_	*h*_0_	*h*_1_	*h*_2_	*q_c_*_1_	*T_c_*_1_	*q_c_*_2_	*T_c_*_2_	*q_c_*_3_	*T*_3_
**All**	$3.29\times 10^{-07}$	$5.09\times 10^{-16}$	$6.21\times 10^{-05}$	$1.55\times 10^{-06}$	$3.77\times 10^{-213}$	$3.32\times 10^{-01}$	$4.36\times 10^{-02}$	$4.34\times 10^{-03}$	$5.00\times 10^{+00}$	$1.71\times 10^{-09}$	$8.56\times 10^{+02}$
**9**	$5.83\times 10^{-08}$	$4.58\times 10^{-06}$	$5.03\times 10^{-05}$	$3.87\times 10^{-07}$	$6.34\times 10^{-130}$	$1.07\times 10^{-01}$	$3.73\times 10^{-02}$	$2.11\times 10^{-06}$	$4.63\times 10^{+01}$	$1.38\times 10^{-03}$	$6.08\times 10^{+01}$
**8**	$4.45\times 10^{-08}$	$5.56\times 10^{-17}$	$4.01\times 10^{-05}$	$7.38\times 10^{-30}$	$4.85\times 10^{-88}$	$6.96\times 10^{-03}$	$7.22\times 10^{-01}$	$1.20\times 10^{-03}$	$5.00\times 10^{+00}$	$2.27\times 10^{-04}$	$1.00\times 10^{+03}$
**6**	$9.82\times 10^{-08}$	$3.03\times 10^{-06}$	$7.73\times 10^{-05}$	$1.88\times 10^{-11}$	$6.89\times 10^{-10}$	$9.39\times 10^{-02}$	$4.76\times 10^{-02}$	$1.89\times 10^{-03}$	$2.42\times 10^{+01}$	$8.69\times 10^{-08}$	$4.44\times 10^{+02}$
**5**	$9.48\times 10^{-08}$	$9.21\times 10^{-06}$	$7.92\times 10^{-05}$	$9.95\times 10^{-07}$	$1.15\times 10^{-24}$	$2.08\times 10^{-01}$	$2.61\times 10^{-02}$	$1.25\times 10^{-03}$	$4.27\times 10^{+01}$	$1.79\times 10^{-03}$	$5.00\times 10^{+01}$
**4**	$1.06\times 10^{-07}$	$2.94\times 10^{-15}$	$7.27\times 10^{-05}$	$3.30\times 10^{-15}$	$4.33\times 10^{-18}$	$2.29\times 10^{-01}$	$2.73\times 10^{-02}$	$2.37\times 10^{-03}$	$5.00\times 10^{+00}$	$2.67\times 10^{-10}$	$5.67\times 10^{+02}$
**3**	$1.93\times 10^{-07}$	$7.36\times 10^{-22}$	$2.37\times 10^{-04}$	$1.02\times 10^{-06}$	$4.67\times 10^{-69}$	$1.74\times 10^{-02}$	$5.80\times 10^{-01}$	$1.75\times 10^{-11}$	$1.03\times 10^{+01}$	$1.14\times 10^{-11}$	$6.74\times 10^{+02}$
**2**	$6.10\times 10^{-07}$	$4.04\times 10^{-15}$	$7.92\times 10^{-04}$	$3.49\times 10^{-06}$	$1.71\times 10^{-12}$	$1.21\times 10^{-02}$	$1.00\times 10^{+00}$	$6.69\times 10^{-03}$	$7.37\times 10^{+00}$	$4.62\times 10^{-03}$	$1.26\times 10^{+02}$

## Appendix B. Supplementary Measurement Allan Deviation

For the verification, because of the camera failure, we repeated the testing steady sequence capture after replacing the failing camera. In Figure A1 we demonstrate ‘z’ dimension of M1 marker for maximal numbers (10) of each camera type and all the cameras together. We could observe that short term correlated noises disappeared, whereas the bumps representing periodic and long-term correlated noises remain.
